# Development of sensitive ddPCR assays to reliably quantify the proviral DNA reservoir in all common circulating HIV subtypes and recombinant forms

**DOI:** 10.1002/jia2.25185

**Published:** 2018-09-14

**Authors:** Kobus J Bosman, Annemarie MJ Wensing, Aster E Pijning, Wilco J van Snippenberg, Petra M van Ham, Dorien MC de Jong, Andy IM Hoepelman, Monique Nijhuis

**Affiliations:** ^1^ Department of Medical Microbiology University Medical Center Utrecht Utrecht the Netherlands; ^2^ Department of Internal Medicine and Infectious Diseases University Medical Center Utrecht Utrecht the Netherlands

**Keywords:** human immunodeficiency virus, subtypes, reservoir, cure, quantification, digital PCR

## Abstract

**Introduction:**

The latent reservoir is the main barrier on the road to HIV cure, and clinical approaches towards eradication are often evaluated by their effect on proviral DNA. To ensure inclusiveness and representativeness in HIV cure studies, proviral DNA quantification assays that are able to detect all common circulating HIV clades are urgently needed. Here, three HIV DNA assays targeting three different genomic regions were evaluated for their sensitivity and subtype‐tolerance using digital PCR.

**Methods:**

A subtype‐B‐specific assay targeting *gag* (GAG) and two assays targeting conserved sequences in *ltr* and *pol* (LTR and JO) were assessed for their sensitivity and subtype‐tolerance in digital PCR (Bio‐Rad QX200), using a panel of serially diluted subtype reference plasmids as well as a panel of clinical isolates. Both panels represent subtypes A, B, C, D, F, G and circulating recombinant forms (CRFs) AE and AG, which together are responsible for 94% of HIV infections worldwide.

**Results:**

HIV subtype was observed to greatly affect HIV DNA quantification results. Robust regression analysis of the serially diluted plasmid panel showed that the GAG assay was only able to linearly quantify subtype B, D and G isolates (4/13 reference plasmids, average *R* ^2^ = 0.99), whereas LTR and JO were able to quantify all tested isolates (13/13 reference plasmids, respective average *R* ^2^ = 0.99 and 0.98). In the clinical isolates panel, isolates were considered detectable if all replicates produced a positive result. The GAG assay could detect HIV DNA in four out of five subtype B and one out of two subtype D isolates, whereas the LTR and JO assays detected HIV DNA in all twenty‐nine tested isolates. LTR and JO results were found to be equally precise but more precise than GAG.

**Conclusions:**

The results demonstrate the need for a careful validation of proviral reservoir quantification assays prior to investigations into non‐B subtype reservoirs. The LTR and JO assays can sensitively and reliably quantify HIV DNA in a panel that represents the worldwide most prevalent subtypes and CRFs (A, B, C, D, AE, F, G and AG), justifying their application in future trials aimed at global HIV cure.

## 1 Introduction

The HIV‐1 pandemic is countered using antiretroviral drugs that intervene in several steps of the viral life cycle. Effective treatment drastically reduces and durably suppresses plasma HIV RNA to below the limit of detection of currently available diagnostic assays. HIV DNA levels on the other hand decrease only slightly upon treatment initiation and remain stable for many years despite effective treatment 1. This reservoir of HIV DNA is the source of rebound viraemia if antiretroviral therapy (ART) is interrupted. It requires patients to undergo lifelong ART treatment and is the main focus of global HIV eradication efforts 2, 3, 4. The potency of candidate cure treatments can be evaluated by their effect on the levels of HIV DNA. Indeed, many studies rate the success of candidate cure treatments using quantitative PCR with assays of primers and probes that match a small target sequence within the HIV genome 5, 6, 7, 8, 9, 10, 11, 12, 13, 14.

An assortment of assays has been developed to estimate total HIV DNA based on the abundances of, for example, *ltr* 15, 16, 17, 18, 19, the junction between *ltr* and *gag* 20, 21, *gag* 22, 23, 24 and *pol* 19, 25, but this diversity of HIV DNA assays raises three major concerns. Firstly, in order to strive towards global HIV cure, research should be optimized to detect all common circulating HIV clades. The main focus of clinical research has been subtype B, as this is the most prevalent subtype in research‐rich countries. Yet, worldwide prevalence of subtype B is only 11% and some reports indicate that subtype diversity is shifting in traditionally subtype B countries 26, 27. HIV DNA quantification assays that are specifically geared towards subtype B are prone to complicate the monitoring of non‐B subtype reservoirs. Secondly, a high variability in HIV subspecies may co‐exist even within a single patient 28, 29, 30, 31. Assays that selectively detect subspecies may skew quantification outcomes. Thirdly, recent evidence suggests that a large fraction of HIV DNA found in effectively treated patients is comprised of incomplete HIV genomes 32, 33, 34. Recent evidence suggests that deletion or preservation of specific genome segments is not random, and therefore not all target sequences may correctly represent clinically relevant HIV DNA load 35. Together, these considerations underline the risk of relying on an assay that targets a small non‐conserved part of the HIV genome. Such an assay may incorrectly gauge the effect of novel therapeutics on the HIV DNA reservoir and thereby confound the clinical relevance of global interventions aimed at HIV cure.

A universal subtype‐tolerant assay for the quantification of HIV DNA is urgently needed to ensure the interpretability, repeatability and comparability of future global HIV cure trials. In this study, we compared three HIV DNA assays in the Bio‐Rad QX200 ddPCR platform (Bio‐Rad, Hercules, CA, USA) for their quantification performance on a panel of isolates that together represent 94% of HIV‐1 group‐M subtype variation worldwide. We demonstrate the variation‐intolerance of the subtype‐B‐specific GAG assay, and present two sensitive assays that target the *pol* and *ltr* regions and are capable of quantifying HIV DNA in all tested group‐M subtypes and circulating recombinant forms.

## 2 Methods

### 2.1 Generation of subtype reference plasmids

We have generated a subtype reference plasmid panel representing a wide range of HIV subtypes from different countries of origin. We used a previously published full‐length molecular clone from a primary isolate belonging to the CRF02‐AG group of recombinant viruses that was used directly for transformation 36. All other reference plasmids were cloned to contain the 5′ part of the HIV genomes, encompassing the primer binding sites of the three tested assays. The isolates to be cloned were chosen from a panel of virus isolates consisting of diverse subtypes and CRFs (BBI Biotech Research Laboratories Inc.). Clones were constructed from two subtype A, two subtype B, two subtype C, two subtype D, two subtype AE, one subtype F and one subtype G virus isolate (Table S1). Together with the subtype AG clone, this panel of clones represents 94% of worldwide HIV‐1 subtypes 37. The subtype reference plasmids were sequenced by Sanger sequencing and the LTR, JO and GAG target sequences were submitted to the Los Alamos National Laboratory sequence database website QuickAlign tool and aligned against the LANL HIV1 Complete Nucleotide Filtered web alignment (http://www.hiv.lanl.gov/). The results were summarized by subtype or CRF and for each plasmid the sequence variants of the corresponding subtype or CRF were extracted. A graphical overview of the representativeness of the subtype plasmids for the worldwide intra‐subtype variation is given in Figure S2.

In order to construct the subtype reference plasmids, RNA was isolated from the viral reference culture supernatants with the Boom extraction method 38 and used for RT‐PCR with Superscript‐III One‐Step Platinum Taq (Invitrogen, Carlsbad, CA, USA) and nested PCR with Platinum Taq DNA Polymerase High Fidelity (Thermo Fisher Scientific, Waltham, MA, USA) using isolate‐specific primers (Tables S2–S4). Amplicons were purified from PCR reactions with Qiaquick PCR Purification kit (Qiagen, Hilden, Germany), A‐tailed with DreamTaq DNA polymerase (Thermo Fisher Scientific), again purified with the Qiaquick PCR purification kit (Qiagen), ligated into vector pGEM‐T Easy (Promega, Madison, WI, USA) using T4 DNA ligase (New England Biolabs, Ipswich, MA, USA) and again purified with the Qiaquick PCR Purification kit (Qiagen).

*Escherichia coli* JM109 High Efficiency Competent Cells (Promega) were used for transformation, colonies were picked and cultured overnight at 37°C in 5 mL LB medium supplemented with ampicillin (40 μg/mL) and plasmids were isolated using the Qiaprep Spin Miniprep Kit (Qiagen).

Plasmids were digested using *Eco*RI and Buffer H (Roche, Basel, Switzerland) prior to diluting, in order to ensure the repeatability of the dilutions. The underlying mechanism remains speculative but perhaps linearization prevents coiled plasmids from clumping together and thereby improves homogeneous mixing and diluting. Digested plasmids were purified with the Qiaquick PCR Purification kit (Qiagen) and DNA concentrations were determined with the Qubit dsDNA HS Assay kit (Invitrogen). Plasmid‐specific backbone and insert sizes were used to calculate the molecular weight of each plasmid using the formula An × 313.2 + Tn × 304.2 + Cn × 289.2 + Gn × 329.2 + 79 with An, Tn, Cn and Gn denoting the total number of nucleotides present in each plasmid 39. Plasmid‐specific molecular weights were used to calculate copy numbers from DNA concentrations and used to dilute the DNA to contain 3750 copies/μL, after which this concentration was diluted stepwise to contain 750, 150, 30, 6, 1.2 and 0.24 copies/μL. Note that concentrations were chosen 20% higher to account for the same rate of dilution that is later applied by pre‐ddPCR *Eco*RI restriction. Per dilution, 3 μL was supplemented with 12 μL of 300 ng/μL HIV‐negative donor peripheral blood mononuclear cell (PBMC) DNA.

### 2.2 Patient samples

Patient material was obtained through the Dutch HIV Monitoring Foundation [Stichting HIV Monitoring (SHM)], which is paid for by the Dutch government and has registered all HIV‐infected patients in a nationwide cohort. This cohort is known as the AIDS Therapy Evaluation in the Netherlands (ATHENA) cohort. Data and samples obtained from patients in clinical care in or after 1996 in any of the 27 HIV treatment centres in the Netherlands have been systematically collected as part of routine care 40. Patients can choose to be excluded from prospective data and sample collection in ATHENA. Up to and including 31 December 2016, a total of 25,564 persons with HIV infection were registered through the Dutch HIV treatment centres by SHM of whom 97.9% had consented to data and sample collection 41, 42. Through the ATHENA cohort, PBMCs are only collected at day of diagnosis. PBMCs were used from four patients infected with subtype A, five patients with subtype B, three patients with subtype C, two patients with subtype D, four patients with subtype F, three patients with subtype G, four patients with CRF AE and four patients with CRF AG. Patient PBMCs were isolated from fresh blood by Ficoll gradient centrifugation and stored at −80°C as dry pellets of 5 million cells each until further processing.

### 2.3 Control samples

Water was used as a no template control (water NTCs) and DNA isolated from the PBMCs of HIV‐negative healthy donors was used as a DNA template control (PBMC DNA NTCs). DNA isolated from the U1 cell line was used as a positive control (NIH AIDS Reagent Program).

### 2.4 DNA extraction

Total DNA was extracted from U1 cells and HIV+ and HIV− PBMCs using the DNeasy mini kit (Qiagen). HIV+ PBMC DNA eluates with a concentration of greater than 240 ng/μL were diluted to the said concentration in order to avoid overloading the ddPCR reactions (1 μg DNA maximum after digestion).

### 2.5 Digital PCR

Digital PCR was performed in the QX200 Droplet Digital PCR system (Bio‐Rad), further referred to as the QX200. Fifteen microlitres of each sample was digested for one hour at 37°C using 1.8 μL of 10x Buffer H (Roche) and 1.2 μL *Eco*RI (10 U/μL, Roche). From the digestion mixture, 15 μL was mixed with 51 μL of a mastermix containing 33 μL ddPCR Supermix for probes (no dUTP) (Bio‐Rad), primers and probe and demineralized water. Droplets were created using the Droplet Generator and 70 μL of oil per sample, and each well of a 96‐well plate was supplied with 20 μL of the final reaction. PCR was performed in a T100 thermal cycler (Bio‐Rad) with universal, manufacturer‐recommended cycling protocol and droplets were read using a QX200 droplet reader. Samples were only considered if more than 10,000 droplets were read.

### 2.6 Threshold setting

Because the choice of threshold can severely affect quantification outcome, a comparison was made between manual thresholding, thresholds based on the standard deviation of the negative cloud and ddpcRquant. Based on this comparison, ddpcRquant was used to set the thresholds in this study and all droplets above these thresholds were considered positive in the analyses (Data S1, Tables S5 and S6).

### 2.7 Assays

There are many available assays that have been reported and used to quantify HIV DNA, of which most assays target the *ltr* 16, 17, 19, 20, 21, 43, 44, 45, 46, 47, 48, 49, 50, 51, 52, 53, 54, 55, 56, 57, 58 *, gag* 22, 23, 43, 44, 54, 56, 59, 60, 61, 62 *,* or *pol* 19, 25, 58, 60, 63, 64, 65, 66, 67 regions 68. To the best of our knowledge, we queried the target sequence of the most prominent assays to all known subtype and CRF entries in the LANL HIV1 Complete Nucleotide Filtered web alignment (http://www.hiv.lanl.gov/). The analysis shows that target sequences of assays targeting the *ltr* 16, 20, 47, 52, 55 and *pol* 25, 63, 64 regions are rather conserved, whereas target sequences of assays in the *gag* 22, 23, 56, 69 region are relatively less conserved (Figure S1). Notably, assays within the same genomic region demonstrate comparable complementarity profiles, warranting the selection of one representative assay per genomic region. The gag1 assay that targets a region in *gag*, further referred to as the GAG assay, was used with previously published primers and probe 22. An unnamed assay targeting *pol* that was first described by Rousseau et al. without specification of reporter or quencher was used in our study with a FAM reporter and a ZEN quencher (see Data S2 for the reason for selecting the ZEN quencher) and will be referred to as the JO assay 63, 70, 71. An unnamed assay targeting *ltr,* further referred to as the LTR assay, was used as previously reported 52. Universal/manufacturer‐recommended concentrations were used for all forward and reverse primers (900 nmol/L) and all probes (300 nmol/L), which yielded good PCR efficiency as can be deduced from the robust regression trend lines in the subtype reference plasmid dilution experiments (Figure 5).

## 3 Results

### 3.1 Complementarity

Complementarity of the primers and probes to the plasmid target sequences was evaluated by Sanger sequencing of the subtype plasmids (Table S7A–C). Of the three assays, the JO assay is most complementary to the subtype reference plasmid panel with no mismatches to the forward and reverse primers and only a single mismatch in the probe target sequence of A‐clone 1, both C‐clones and the F‐ G‐ and AG‐clones. For the mix of reverse primer variants of the LTR assay, mismatches were scored by counting the number of mismatches with the best matching variant only. The LTR assay is less complementary than the JO assay, as the forward primer showed a single mismatch with A‐clone 1, AE‐clone 1 and the G‐clone, the probe had a single mismatch with the probe sequence in A‐clone 1 and AE‐clone 1 and two mismatches with AE‐clone 2, and the best‐matching reverse primer had a single mismatch with A‐clone 1, B‐clone 1, D‐clone 1 and AE‐clone 1. The GAG assay demonstrated most mismatches, with its forward primer having a mismatch abundance of one mismatch (B‐clone 1), two mismatches (C‐clone 1, both D‐clones, AE‐clone 1, G‐clone), three mismatches (F‐clone), four mismatches (A‐clone 2, C‐clone 2, AE‐clone 2) and even five mismatches (A‐clone 1, AG‐clone), its probe having a single mismatch in clones C‐clone 2, D‐clone 2, AE‐clone 1, the F‐clone and the AG‐clone and two mismatches in A‐clone 2, C‐clone 1, D‐clone 1 and AE‐clone 2, and its reverse primer having one mismatch (A‐clone 2, both D‐clones and the AG‐clone), two mismatches (both C‐clones, both AE‐clones and the F‐clone) or three mismatches (A‐clone 1). Assay performance in patient isolates was estimated by querying all primers and probes in the Los Alamos National Laboratory sequence database website QuickAlign tool and alignment against the LANL HIV1 Complete Nucleotide Filtered web alignment (http://www.hiv.lanl.gov/). Summarized for all sequences, the percentages of subtype references without any mismatches with the forward primer, probe and reverse primer are 14%, 33% and 27% for GAG, 79%, 57% and 89% for JO and 69%, 47% and (for the two reverse primers cumulatively) 60% in the case of LTR (Figure S3A). Furthermore, specifications for separate subtypes are given in Figure S3B–D.

### 3.2 Specificity

The three assays were scored for their specificity for HIV DNA using positive controls, HIV‐negative donor DNA controls and water controls. All 17 positive controls prepared from DNA of the clonal U1 cell line were highly positive using all three assays, justifying the relevance of the assays for the detection of HIV DNA. Because the DNA of U1 cells contains HIV DNA as well as human genomic DNA, we verified whether the assays are specific for HIV DNA by testing the DNA of HIV‐negative donors (PBMC DNA NTCs). Each assay was used to test fifteen of these PBMC DNA NTCs, of which the LTR and JO assay each tested two samples as positive and the GAG assay tested three samples as positive (Figure S4), an approximate 13% to 20% false‐positive rate. Of note, the few positively tested controls only contained few droplets, indicating that the stochastic positivity in the control samples is unlikely to represent off‐target quantification of endogenous human DNA. In order to further assess the cause of positive droplets in the PBMC DNA NTCs, 22 water NTC samples were tested with each assay and returned a false‐positive rate of 9% to 27% with two, six and two positive samples with the LTR, JO and GAG assay, respectively, excluding that false positivity is caused by endogenous off‐targets in the PBMC DNA NTCs (Figure S4). The ratio between positive and negative outcome of the negative controls was not significantly different between the type of control or assay, as tested using Fisher's exact tests (*p* > 0.05). Concentrations found in negative controls were used to calculate assay‐specific limits of blank (LoB) with LoB = mean_blank_ + 1.645(SD_blank_) 72. The resulting LoB is 1.72, 1.85 and 1.94 target copies for the LTR, JO and GAG assays, respectively. These results suggest that the three assays are equally capable of detecting HIV DNA and do not have off‐targets in the human genome but, as reported for all assays used in digital PCR thus far, should be reviewed critically in case of results lower than two copies.

### 3.3 Detectability

A notable property of the subtype plasmids is that each individual plasmid contains the 5′ half of the HIV genome to accommodate one target for each of the three assays. The subtype plasmids were serially diluted and each dilution step was subsequently dispensed into the three assays, allowing for a direct comparison of their quantification results (Table S8). Replicate samples were defined as detected when one or more positive droplet was detected and detectability was investigated by calculating the ratio between the number of detectable and undetectable replicates and differences were assessed for significance (Fisher's exact tests). Detectability of the GAG assay was found equal to the JO assay in the 0.2‐copy and 1‐copy input samples, and equal to the LTR assay in the 0.2‐copy samples. In these low ranges, detectability is likely to be equally stochastic in all three assays due to sampling error of target sequences and the detection of false positives that is inherent in digital PCR. In the samples intended to contain 3125, 625, 125 and 25 target copies, detectability of the LTR and JO assays was found to be equal to each other and significantly better than the GAG assay. The significantly poorer detectability of the GAG assay is evidently affected by its poor performance in most subtype plasmids. The GAG assay was only able to detect the majority of dilution range samples from the two subtype B plasmids, one of the two subtype D plasmids and the subtype G plasmid. Coinciding with the sequencing results discussed above, within the tested panel these are the same plasmids that demonstrate the least number of mismatches with GAG, suggesting that a maximum of four mismatches in primers and probe is allowed whereas five or more mismatches abrogate detectability.

In addition to the controlled approach of subtype plasmid dilution series, PBMC DNA from 29 treatment‐naïve patients diagnosed with a variety of HIV subtypes and CRFs was used to validate the detectability of the three tested assays (Table S9). The GAG, JO and LTR assays, respectively, detected HIV DNA in 28, 86 and 86 out of 87, 87 and 86 reactions, of which 23, 86 and 86 were above the assay‐specific LoB. The total number of detected samples, as well as the number of detected samples above LoB, was significantly higher in the LTR and JO assays compared to the GAG assay (Fisher's exact tests, *p* < 0.001). Notably, whereas the LTR assay produced a positive result in all tested replicates of all twenty‐nine patient isolates and the JO assay missed one replicate of one AG isolate, GAG was only able to detect target in all three replicates of four out of five subtype B isolates and one of the two subtype D isolates. As was the case for the plasmid dilution experiment, these data indicate that the LTR and JO assays are capable of detecting HIV DNA in all isolates from all tested subtypes and CRFs, whereas GAG detectability is restricted to, but not guaranteed for all, subtype B and D isolates.

### 3.4 Quantification outcome

**Figure 1:**
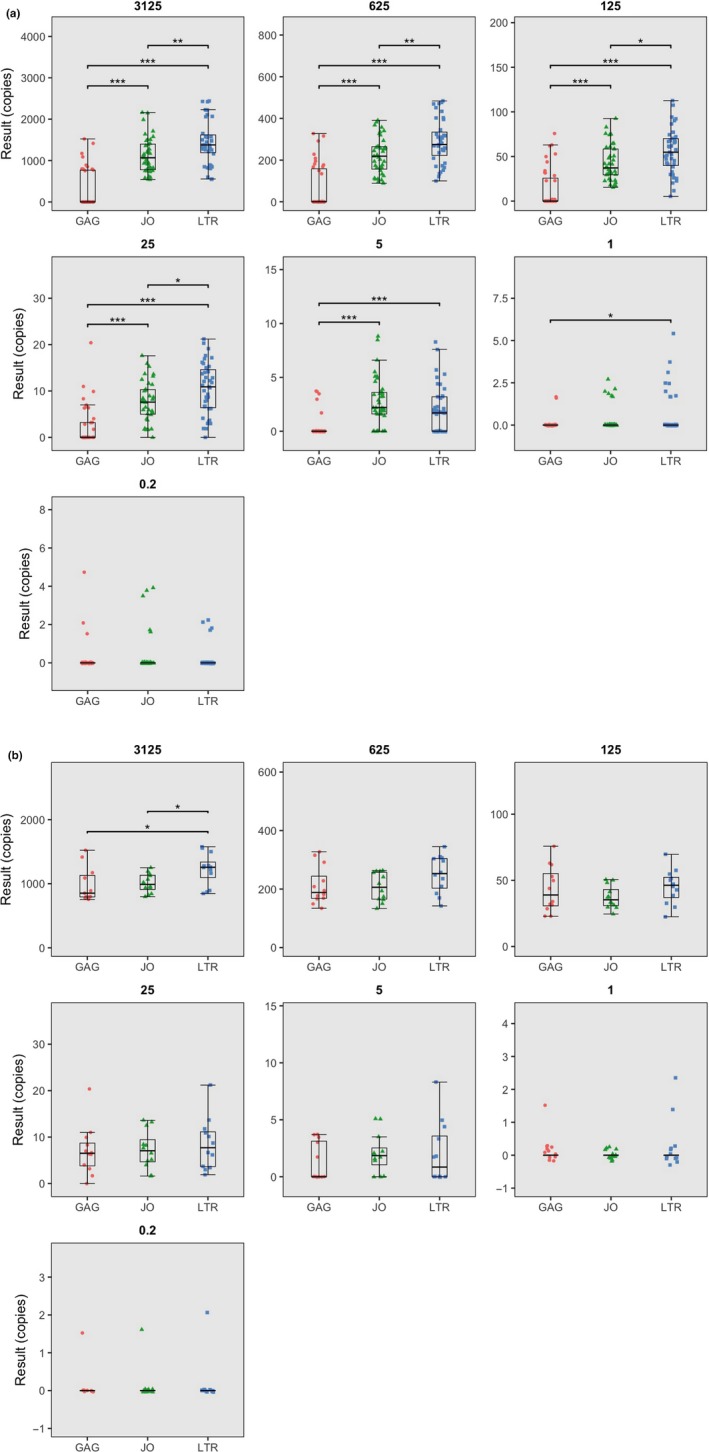
**Boxplots depicting subtype reference plasmid quantification results, stratified per input concentrations.** (**a**) All plasmids combined. (**b**) Only subtype plasmids B‐clone 1, B‐clone 2, D‐clone 2 and the G‐clone.

In order to investigate whether the assays produce similar quantification results, a comparison was made between quantification results and significance of the difference was tested using Welch's *t* test unassuming of sample pairing or variance. In order to differentiate from detectability, assay quantification outcome differences between assays were only compared using subtype plasmids or patient isolates of which the majority of replicates could be detected in both compared assays. In the subtype reference plasmid experiment, all plasmids were therefore included to show that LTR results are higher than JO results in 3125, 625, 125 and 25 copy input samples but equal in 5, 1 and 0.2 copy input samples (Figure 1A). A subset of subtype plasmids B‐clone 1, B‐clone 2, D‐clone 2 and G‐clone was used to compare GAG to JO and LTR. In this subset of plasmids, GAG results proved equal to JO results in all dilutions and equal to LTR results in all but the 3125 input samples where GAG results were lower (Figure 1B). Although it is difficult to decide which assay represents actual plasmid input quantity, in view of the equal false‐positive rates of the assays it seems more likely that the LTR results represent true plasmid input quantity and JO and GAG results are underestimates. These data indicate that, corrected for detectability, the LTR assay produces higher results than both JO and GAG, and that JO and GAG produce equal results. Of note, even most LTR results are lower than the intended target copies. This observation can be attributed to two factors. First, vortexing and freeze‐thaw cycles may have damaged and sheared the target DNA, preventing their PCR amplification and detection. Second, even dilutions that were prepared in parallel from the same stock varied two to threefold from each other (data not shown). This variation is likely introduced when the lowest measurable concentration of digested plasmid DNA is further diluted 10^5^‐fold to obtain the concentration needed to generate the 3215‐input sample.

**Figure 2:**
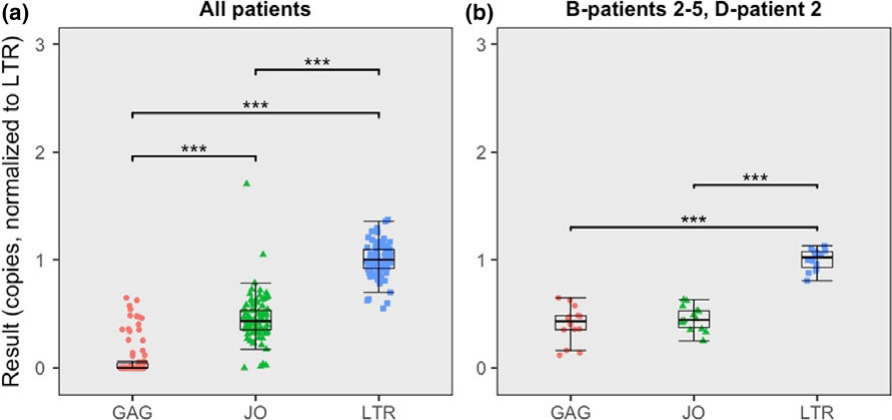
**Boxplots depicting patient isolate quantifications results.** (**a**) All patient isolates combined. (**b**) Only GAG‐detectable patient isolates B‐patient 2, B‐patient 3, B‐patient 3, B‐patient 4, B‐patient 5 and D‐patient 2.

In the patient isolate experiments, isolated DNA was used directly for quantification without any normalization for total amount of DNA tested or size of the reservoir. The quantification output of each patient isolate was therefore normalized for the average isolate‐specific LTR results in order to allow for a comparison of relative assay quantification outcome. As was the case for the plasmid experiments, quantification outcome assessment of the patient isolates is substantially affected by the number of undetectable samples, which we attempted to correct for by only analysing isolates that both compared assays could detect in triplicate. All isolates passed this criterion to show that the results are significantly higher in the LTR assay compared to the JO assay (*p* < 0.001) (Figure 2A). A subset of isolates of which all three replicates were detectable by GAG (four subtype B and 1 subtype D isolate) was further investigated and demonstrated that GAG results were significantly lower than LTR but comparable to JO (Welch's two‐sample t‐tests) (Figure 2B), and these results are in accordance with the subtype reference plasmid dilution series. It should however be noted that as opposed to plasmids, actual target abundances in patient isolates may vary between assays. The LTR assay, for example, finds two target sequences in each intact proviral genome which may explain its twofold higher quantification outcome.

### 3.5 Precision and quantitative linearity

**Figure 3:**
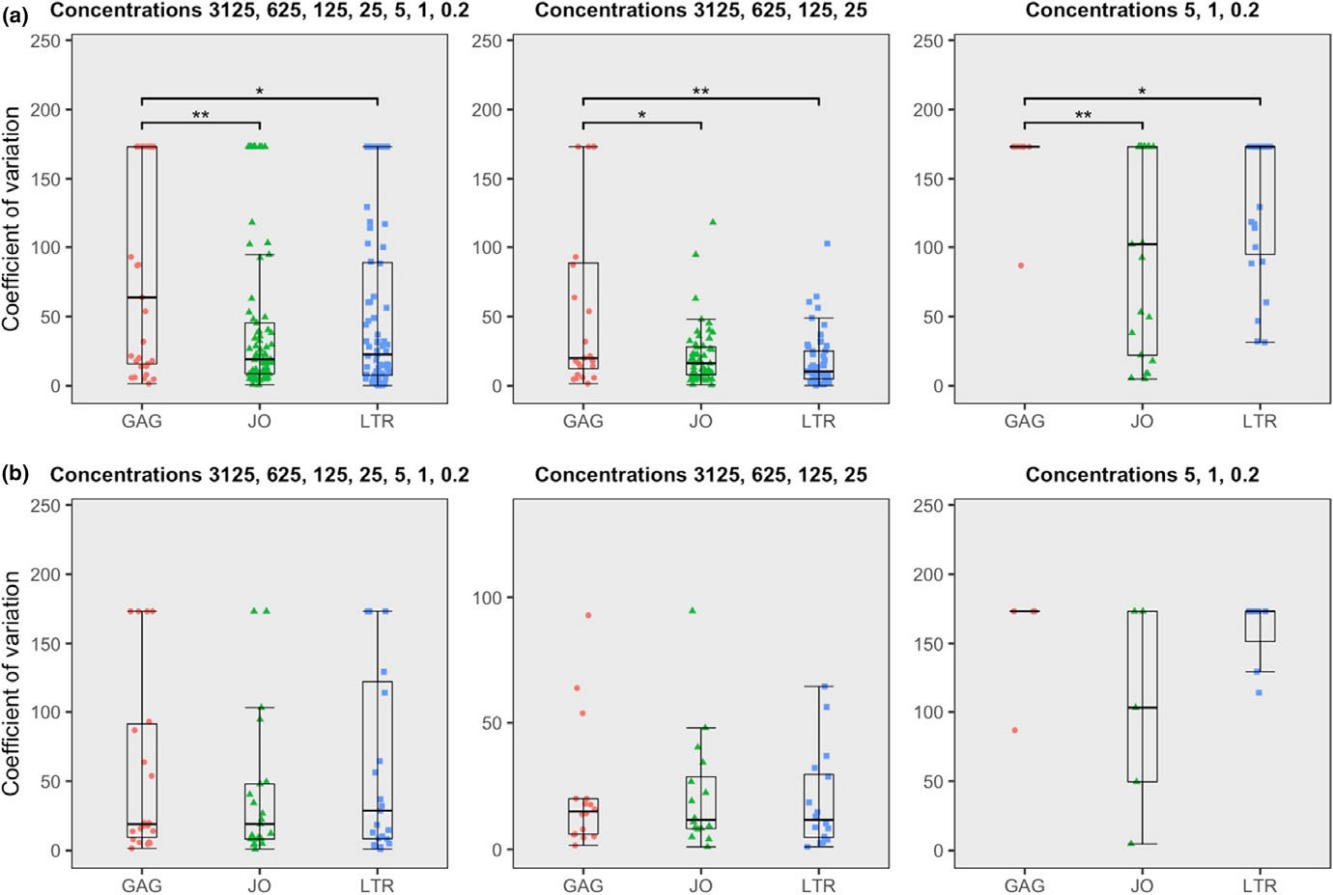
**Boxplots showing the coefficient of variation (CV) for the subtype plasmids, either all input concentrations combined, grouped for the highest input or grouped for the lowest input.** (**a**) All subtype plasmid results combined. (**b**) Only CVs of subtype plasmids B‐clone 1, B‐clone 2, D‐clone 2 and the G‐clone.

**Figure 4:**
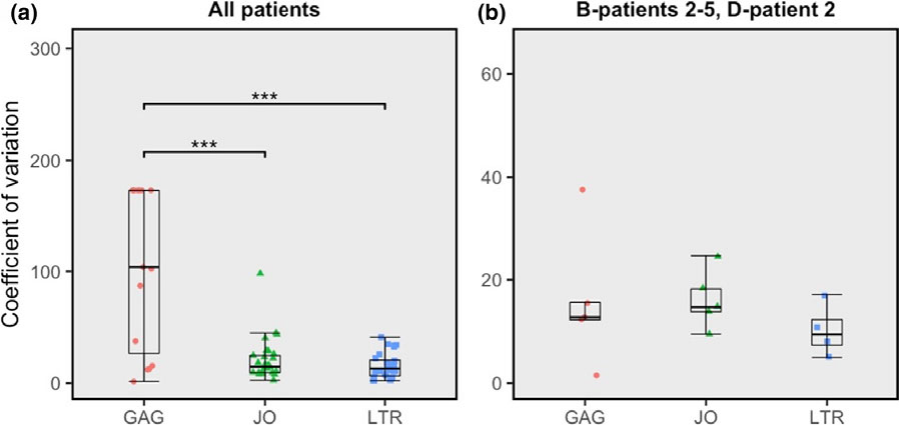
**Boxplots showing the coefficient of variation (CV) for the patient isolates.** (**a**) All patient isolate results combined. (**b**) Only GAG‐detectable patient isolates B‐patient 2, B‐patient 3, B‐patient 4, B‐patient 5 and D‐patient 2.

In addition to detectability and quantification outcome, assay dependability was scored for precision and quantitative linearity. Similar to the comparison of quantification outcome, assay dependability estimates were only compared using subtype plasmids of which the majority of samples could be quantified in both compared assays. First, precision was determined by calculating coefficients of variation (CV), defined as the percentage of standard deviation from the mean. All subtype plasmids combined show that the LTR and JO assays are equally precise but more precise than GAG, either for all concentrations combined or grouped for the four highest or three lowest concentrations (Figure 3A). The subset of plasmids B‐clone 1, B‐clone 2, D‐clone 2 and the G‐clone demonstrates that in those plasmids, all three assays are equally precise (Welch *t* tests) (Figure 3B). The precision of the patient sample quantifications was evaluated by calculating the CV after normalization to the average isolate‐specific LTR results. In accordance with the conclusion from the subtype reference plasmid quantifications, the LTR and JO assays were found to be equally precise in all patient isolates and more precise than GAG (Figure 4A), whereas all assays proved equally precise in isolates that were detected in all three replicates by the GAG assay (Figure 4B).

**Figure 5:**
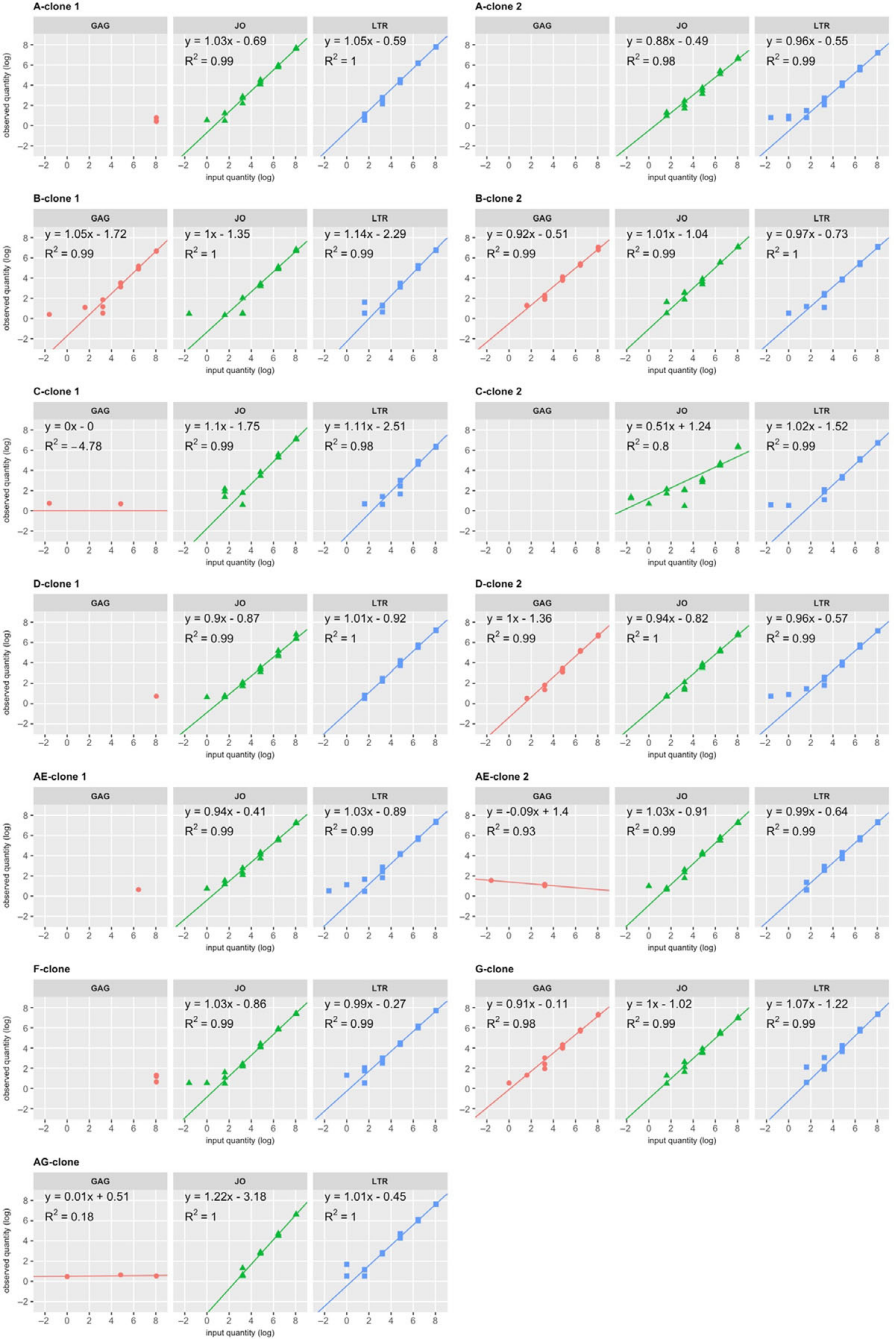
Robust regression fits to the subtype reference plasmid dilution series.

Quantification results of the plasmid dilutions were fit to a robust regression model (Figure 5). In accordance with detectability, the model could be fit to the LTR and JO assay quantifications of all subtype reference plasmids and to the GAG assay quantifications only of plasmids B‐clone 1, B‐clone 2, D‐clone 2 and the G‐clone. For LTR, JO and GAG, respectively, the robust regression fits demonstrate an average linearity of *R* ^2^ = 0.99, 0.98 and 0.99 which was not significantly different between the assays (Welch's two‐sample *t* tests). Efficiency was derived from the slope of the robust regression trend lines and found to be between 90% and 122% with no distinct differences between assays or subtype reference plasmids, indicating that PCR efficiencies for all three assays are adequate and equivalent. Together, these data indicate that precision, quantitative linearity and efficiency are equal between LTR and JO in all subtype plasmids, and equal between LTR, JO and GAG in the subtype plasmids that GAG could detect.

## 4 Discussion

This study demonstrates that the choice of assay can severely affect ddPCR quantification results, and that the LTR and JO assays are able to reliably quantify HIV DNA in a panel of isolates representing the subtypes that together are responsible for 94% of worldwide HIV‐1 group‐M infections.

Assays that perform well on one subtype may not necessarily be capable of detecting other HIV subtypes and CRFs. Indeed, whereas the GAG assay is able to sensitively quantify complementary subtype B, D and G plasmids and isolates, it fails to detect even the highest input samples of other subtypes and CRFs with which it bears more mismatches. The LTR and JO primer and probe sequences are highly conserved within the LANL HIV1 Complete Nucleotide Filtered web alignment (Figure S3A–D) and are able to detect their target in all tested subtypes and CRFs. These observations are in line with earlier studies demonstrating that single internal mismatches may reduce but not abrogate PCR efficiency 73, 74, whereas PCR amplification may fail and lead to false‐negative results if mismatches are too abundant or located on the 3′ terminal end of primers 75, 76, 77, 78. In total, we have tested over 40 clinical isolates and reference strains of which the latter represent the most common sequence variants in the LANL HIV1 Complete Nucleotide Filtered web database (Figures S2A–C). The fact that the LTR and JO assays target conserved genomic regions where variation is restricted, the representativeness of the subtype reference plasmids and the fact that HIV DNA was detected in all patient isolates without exception emphasize the reliability of the LTR and JO assays.

Reliable quantification of the variable HIV genome demands that all available target sequences are amplified and detected. One parameter by which to assess amplification reliability is precision, which is represented by CV (Figure 3A, Tables S8 and S9). The CVs reported here for LTR and JO are inversely correlated with target abundance and comparable to other reports 14, 19, 22, 62, 79, 80, 81. The precision of the GAG assay is very poor in the subtype reference plasmid dilution experiments as well as in the patient samples, and is clearly affected by undetectable samples in both experiments. Indeed, another important parameter to assess amplification reliability is by comparing assay quantification results, as every single plasmid contains one target for each of the assays. The GAG assay clearly suffers from the multitude of mismatches in most subtype reference plasmids, disallowing primer/probe annealing and PCR amplification. The JO assay was also found to produce significantly lower results than the LTR assay, which is remarkable considering that the LTR and JO assays demonstrate comparable efficiency as described earlier (Figure 5). This contradiction leads to suspect that LTR and JO assay result differences are affected by something other than PCR efficiency. ddPCR allows for a closer inspection of the distribution of droplet fluorescence intensities showing that the positive fraction of droplets is lower and more widely distributed in JO, to the point where it notably merges with the negative fraction in some plasmids (Figure S5). This cloud separation distance appears to affect quantification outcome, as the ratio between LTR‐ and JO‐positive cloud heights is significantly correlated with the ratio between LTR and JO quantification results (robust regression, *p* = 0.011) (Figure S6). It has previously been suggested that digital PCR is tolerant to suboptimal PCR efficiency 19, 62, 79, 81, 82, 83, 84, but our observation demonstrates that factors additional to PCR efficiency determine cloud separation distance and thereby quantification outcome. Although the identity of these factors remains speculative (Data S2), our observations point out the necessity of optimizing PCR efficiency as well as cloud separation distance of digital PCR assays in order to detect all available target molecules.

In patient isolates, another layer of complexity is added, namely that the actual presence of target molecules may vary. It has previously been shown that the majority of proviral genomes is defective 33, 34, 85 and it is currently unknown if deletion or preservation of specific genome segments is entirely random. Although defectiveness of individual proviral genomes cannot be estimated in this study, the average twofold difference between LTR and JO results suggests that the *ltr* and *pol* regions are deleted or conserved in equal proportions. Compared to JO, GAG results are much lower in most isolates. However, GAG and JO results are equal in subtype B patient isolates, indicating that differences between GAG and JO in non‐B subtypes are more likely caused by poor performance of the GAG assay rather than by actual differences in *gag* and *pol* abundances. These data suggest that, on average, there does not seem to be preferential preservation or deletion of the three regions that we tested. It should, however, be noted that the patient isolates that we used for this study were sampled from treatment‐naïve patients with unknown times spent untreated, while preferential preservation may differ for other genomic regions, cell types, times spent untreated and time spent on treatment 13, 33, 35, 85, 86.

Single amplicon assays, for example, by digital PCR are not adequate methods to detect and quantify intact and/or replication competent provirus. Nevertheless, total HIV DNA depletion is used as an important parameter by which to validate the success of candidate cure treatments 11, 43, 45, 60, 87, 88, 89, 90, 91, 92, 93, 94. One example is investigation of the effect of stem cell transplantations (SCT) on the reservoir. SCT is currently the only available procedure that can drastically reduce HIV DNA levels in humans. Patients who have received SCT are likely to have near‐undetectable HIV DNA reservoirs, and generally only allow for small and infrequent sampling due to their frailty. The same holds true for children that initiated ART directly after birth in order to prevent postnatal mother‐to‐child transmission of HIV infection. In these seronegative children, HIV DNA is the only evidence of potential prenatal HIV infection that can support the decision to continue ART treatment. In these delicate cases, an assay that has the most chance to detect its target is preferred, in which case the twofold more abundant *ltr* region would be the preferred target. It is however not yet clear if the ratio between *ltr* and *pol* that we find in patients investigated in this study is true for all HIV eradication strategies or treatment conditions. The JO assay may therefore prove to be useful as a control by which the findings of the LTR assay may be validated. The false‐positive rates of 13% to 20% for PBMC DNA NTCs and 9% to 27% for water NTCs observed in this study (Figure S4) are higher than reports that use a threshold above the highest droplet in NTCs or subtract the results found in NTCs from sample results 95, 96, 97, 98, 99, 100. They are, however, similar to previous reports that used predetermined or algorithm‐driven thresholds yielding false‐positive rates of 8% 101, 13% 95, 15% 102, 21% 62, 25% 22, 40% 19 and 100% 103, affirming the paradigm of false‐positive detection as the major drawback of digital PCR 14, 79, 80, 81. The primers and probes used in this study can also be applied in qPCR, a platform that may be preferred to ddPCR to discriminate between the presence or absence of HIV DNA 22. In addition, qPCR is more likely to be used than ddPCR in resource‐limited settings, where assays that are able to quantify non‐B subtypes are of particular importance.

## 5 Conclusions

The present results demonstrate that the choice of assay can severely affect ddPCR quantification results. The LTR and JO assays are able to detect HIV DNA regardless of subtype or CRF classification, whereas the GAG assay is only able to detect some, but not all, subtype B, D and G samples. Corrected for detectability, all three assays proved equally precise but the LTR assay produced higher quantification results than the JO and GAG assay. The present results demonstrate the need for careful validation of proviral reservoir quantification assays prior to investigations of non‐B subtype reservoirs. The LTR and JO assays can sensitively and reliably quantify HIV DNA in a panel representing the worldwide most prevalent subtypes, indicating that they are not affected by variability between subtypes and justifying their application in future HIV cure trials.

## Competing interests

The authors have no competing interests to declare.

## Authors’ contributions

KJB, PMvH and MN designed the study. AIMH enabled access to patient samples. KJB, PMvH, AEP, WJvS and DMCdJ performed the experiments. KJB and MN analysed the data. KJB, AMJW and MN wrote the manuscript. All authors reviewed the manuscript.

## Supporting information


**Table S1**. Virus isolates obtained from BBI Biotech Research Laboratories Inc. used for cloning the subtype reference plasmids.
**Table S2.** Primers used for cloning the subtype reference plasmids.
**Table S3.** RT‐PCR cycling parameters used for subtype reference plasmid cloning.
**Table S4.** Nested PCR cycling parameters used for subtype reference plasmid cloning.
**Data S1.** Threshold‐setting considerations.
**Table S5.** Numbers of false‐positive water NTCs depending on threshold algorithm out of 22 water NTCs tested per assay.
**Table S6.** Numbers of positive PBMC DNA NTCs depending on threshold algorithm out of 15 PBMC DNA NTCs tested per assay.
**Figure S1.** Primer and probe sequence complementarity of our assays and other assays found in the literature to LANL HIV1 Complete Nucleotide Filtered web alignment
**Table S7.** Complementarity of the LTR (A), JO (B) and GAG (C) primers and probes to their binding sites in the subtype reference plasmids
**Figure S2.** Representativeness of the subtype reference plasmids for all intra‐subtype variation
**Figure S3.** Primer and probe sequence complementarity of the LTR, JO and GAG assays to the LANL HIV1 Complete Nucleotide Filtered web alignment
**Table S8.** Quantification results of the subtype reference plasmids; numbers represent absolute copies
**Table S9.** Quantification results of the patient isolates; numbers represent absolute copies
**Figure S4.** Droplet plots of PBMC DNA and water NTCs
**Figure S5.** Droplet distributions of detectable LTR, JO and GAG results in the 3125 concentration subtype reference plasmid experiments
**Figure S6.** Correlation between LTR‐ and JO‐positive cloud difference (*X*‐axis) and LTR and JO quantification results difference (*Y*‐axis)
**Data S2.** On the causes of cloud separation distance.
**Figure S7.** Effect of different quenchers on droplet distributions.
