# Supplementary material for: Development of sensitive ddPCR assays to reliably quantify the proviral DNA reservoir in all common circulating HIV subtypes and recombinant forms
Source: J Int AIDS Soc. 2018 Sep 14;21(9):e25185. doi: 10.1002/jia2.25185 (PMC6138437; doi:10.1002/jia2.25185)

**Supplementary Table 1.** Virus isolates obtained from BBI Biotech Research Laboratories Inc. used for cloning the subtype reference plasmids.

| Referred to in this paper | HIV-1 group or subtype | Isolate ID | Country of origin |
| --- | --- | --- | --- |
| A-clone 1 | A | UG275 | Uganda |
| A-clone 2 | A | POC44951 | Thailand |
| B-clone 1 | B | BK132 | Thailand |
| B-clone 2 | B | US2 | USA |
| C-clone 1 | C | P3100 | Ethiopia |
| C-clone 2 | C | ZB18 | Zambia |
| D-clone 1 | D | SE365 | Senegal |
| D-clone 2 | D | UG274 | Uganda |
| AE-clone 1 | CRF01-AE | CM240 | Thailand |
| AE-clone 2 | CRF01-AE | NP03 | Thailand |
| F-clone | F | BZ126 | Brazil |
| G-clone | G | MIKA-'G' | Kenya |

**Supplementary Table 2.** Primers used for cloning the subtype reference plasmids.

| **Name** | **Clones** | **Type** | **5’ position HXB2** | **3’ position HXB2** | **Sequence (5'-3')** |
| --- | --- | --- | --- | --- | --- |
| AP Fw | All | forward  (outer & nested) | 456 | 495 | GTCTCTCTGGTTAGACCAGATCTGAGC |
| AP1-AB Rv | A-clones  B-clones | reverse (outer) | 5548 | 5578 | TCTTCTGGGGCTTGTTCCATCTATCCTCT |
| AP2-AB Rv | A-clones  B-clones | reverse (nested) | 5038 | 5073 | TGCCACACAATCATCACCTGCCATCTGTTTTCCAT |
| AP3-C Rv | C-clones | reverse (outer) | 5370 | 5402 | CTTATGGCAGAGTCTGCAAAACAATCAAAATA |
| AP4-C Rv | C-clones | reverse (nested) | 5275 | 5311 | CCATTCTATGGAGACTCCATGACCCAAATGCCA |
| AP5-D Rv | D-clones | reverse (outer) | 5326 | 5363 | TGAATTAGTTGGTCGCCAGGCCAGGGTCTACTTGTGT |
| AP6-D Rv | D-clones | reverse (nested) | 5275 | 5310 | CTCCCTGACCCAGATGCCA |
| AP7-F Rv | F-clone | reverse (outer) | 5467 | 5498 | GTTAGTGCCAAATATTGTAGGGATCCT |
| AP8-F Rv | F-clone | reverse (nested) | 5282 | 5311 | CCTCCATTCTATGGAGACTCCCTG |
| AP9-G Rv | G-clone | reverse (outer) | 5175 | 5208 | TCCTAGTGGGATGTGTACTTCTGAACTTACTTT |
| AP10-G Rv | G-clone | reverse (nested) | 5038 | 5074 | CTGCCACACAATCATCACCTGC |
| AP11-AE Rv | AE-clones | reverse (outer) | 5326 | 5365 | CAGTTGGTCTGCTAGGTCAGGATC |
| AP12-AE Rv | AE-clones | reverse (nested) | 5260 | 5297 | CAATTGCCAGTCCTTTTCTCCTG |

**Supplementary Table 3.** RT-PCR cycling parameters used for subtype reference plasmid cloning.

| **Cycles** | **Temperature (°C)** | **Time** |
| --- | --- | --- |
| 1 | 95 | 2 min |
| 10 | 95 | 30 seconds |
|  | 65, -1°C per cycle | 30 seconds |
|  | 68 | 4 minutes |
| 30 | 95 | 30 seconds |
|  | 60 | 30 seconds |
|  | 68 | 4 minutes |
| 1 | 68 | 10 minutes |

**Supplementary Table 4.** Nested PCR cycling parameters used for subtype reference plasmid cloning.

| **Cycles** | **Temperature (°C)** | **Time** |
| --- | --- | --- |
| 1 | 94 | 2 minutes |
| 30 | 94 | 15 seconds |
|  | 60 | 30 seconds |
|  | 68 | 5 minutes |
| 1 | 68 | 5 minutes |

**Supplementary Text 1.** Threshold setting considerations.

First, the algorithms were used to calculate thresholds on positive controls of U1 cell line DNA. None of the results were found to be significantly different between manual thresholding, thresholding based on the standard deviation of the negative cloud or thresholding using ddpcRquant (Welch t-tests). Applied to the negative controls, ddpcRquant thresholds were found to result in the least false-positives and ddpcRquant was therefore used as the algorithm for the data-driven calculation of thresholds on our data (Supplementary Tables 5-6). It should however be noted that for this paper, ddpcRquant was used under two important conditions. The first condition is that PBMC DNA NTCs were used for calibration of the ddpcRquant-threshold. The choice of NTCs used to train the algorithm was found to affect the resulting thresholds. DdpcRquant uses the distribution of droplet intensities of NTCs to calculate block-specific maximum values and feed this to a generalized extreme value model. Therefore, if the narrow droplet distribution of water NTCs is used, the resulting sample thresholds are lower compared to when the wider distribution of donor PBMC DNA NTCs is used. PBMC DNA NTCs were chosen to calibrate ddpcRquant in this paper because the distribution of droplet intensities of actual samples is better resembled by PBMC DNA NTCs than by water NTCs and because ddpcRquant calibrated on PBMC DNA NTCs yielded least false-positives in the negative controls (Supplementary Tables 5-6). The second condition under which ddpcRquant was used is that we used a modified version of the R-script, as we found the R-script that was published alongside the research paper to contain a few incongruities to the theorem described in the paper. After consulting the authors of ddpcRquant an adapted version of the script that harmonizes with the paper was used for the present project (available upon request).

**Supplementary Table 5.** Numbers of false-positive water NTCs depending on threshold algorithm out of 22 water NTCs tested per assay.

| **Method** | **LTR** | **JO** | **GAG** | **Total** |
| --- | --- | --- | --- | --- |
| Manual thresholds (resp. 4000, 1000 and 4000) | 1 | 5 | 1 | 7 |
| Standard-deviation-based thresholding (Dreo et al. 2014) | 5 | 4 | 3 | 12 |
| ddpcRquant, calibrated on water NTCs, original script | 3 | 6 | 2 | 11 |
| ddpcRquant, calibrated on PBMC DNA NTCs, original script | 2 | 11 | 1 | 14 |
| ddpcRquant, calibrated on water NTCs, adapted script | 3 | 6 | 3 | 12 |
| ddpcRquant, calibrated on PBMC DNA NTCs, adapted script | 2 | 6 | 2 | 10 |

**Supplementary Table 6.** Numbers of positive PBMC DNA NTCs depending on threshold algorithm out of 15 PBMC DNA NTCs tested per assay.

| **Method** | **LTR** | **JO** | **GAG** | **Total** |
| --- | --- | --- | --- | --- |
| Manual thresholds (resp. 4000, 1000 and 4000) | 2 | 2 | 3 | 7 |
| Standard-deviation-based thresholding (Dreo et al. 2014) | 3 | 2 | 7 | 12 |
| ddpcRquant, calibrated on water NTCs, original script | 8 | 2 | 6 | 16 |
| ddpcRquant, calibrated on PBMC DNA NTCs, original script | 2 | 4 | 3 | 9 |
| ddpcRquant, calibrated on water NTCs, adapted script | 7 | 2 | 7 | 16 |
| ddpcRquant, calibrated on PBMC DNA NTCs, adapted script | 2 | 2 | 3 | 7 |

Supplementary Figure 1. Primer and probe sequence complementarity of our assays and other assays found in literature to LANL HIV1 Complete Nucleotide Filtered web alignment. Summarize for all sequences for *ltr* assays (A), *gag* assays (B) and *pol* assays (C).

Figure headings indicate assay names of the LTR assay used in this study (Aitken et al. 2013), Ru5 (De Spiegelaere et al. 2014), U (Zack et al. 1990), MH53 (Butler et al. 2011), NEC (Viard et al. 2004), the JO assay in this study (Rousseau et al. 2004), MF30 (Althaus et al. 2010), iSCA (Cillo et al. 2013), the GAG assay used in this study (Bosman et al. 2015), palmergag (Palmer et al. 2013), usRNA (Pasternak et al. 2008) and ZackGAG (Benkirane et al. 1993).

A

B

C

**Supplementary Table 7.** Complementarity of the LTR (**A**), JO (**B**) and GAG (**C**) primers and probes to their binding sites in the subtype reference plasmids. Primers are shown 5’ to 3’, green and red indicate matches and mismatches with the primer.

1. **LTR assay**

|  | HIV-LTR S4 | HIV-LTR-FAMMGB | 3’UNI-KS-6 | 3’UNI-KS-6-AG |
| --- | --- | --- | --- | --- |
| Primer | AAGCCTCAATAAAGCTTGCCTTGA | TAGTGTGTGCCCGTCTG | GAGGGATCTCTAGTTACCAGAGTCACA | GAGGGATCTCTAGTTACCAGAGTCCTA |
|  |  |  |  |  |
| HXB2 | AAGCCTCAATAAAGCTTGCCTTGA | TAGTGTGTGCCCGTCTG | GAGGGATCTCTAGTTACCAGAGTCACA | GAGGGATCTCTAGTTACCAGAGTCACA |
| A-clone 1 | AAGCCCCAATAAAGCTTGCCTTGA | CAGTGTGTGCCCGTCTG | GAGGGATCTCTAGTTACCAGAGTCATA | GAGGGATCTCTAGTTACCAGAGTCATA |
| A-clone 2 | AAGCCTCAATAAAGCTTGCCTTGA | TAGTGTGTGCCCGTCTG | GAGGGATCTCTAGTTACCAGAGTCACA | GAGGGATCTCTAGTTACCAGAGTCACA |
| B-clone 1 | AAGCCTCAATAAAGCTTGCCTTGA | TAGTGTGTGCCCGTCTG | GAGGGATCTCTAGCTACCAGAGTCACA | GAGGGATCTCTAGCTACCAGAGTCACA |
| B-clone 2 | AAGCCTCAATAAAGCTTGCCTTGA | TAGTGTGTGCCCGTCTG | GAGGGATCTCTAGTTACCAGAGTCACA | GAGGGATCTCTAGTTACCAGAGTCACA |
| C-clone 1 | AAGCCTCAATAAAGCTTGCCTTGA | TAGTGTGTGCCCGTCTG | GAGGGATCTCTAGTTACCAGAGTCACA | GAGGGATCTCTAGTTACCAGAGTCACA |
| C-clone 2 | AAGCCTCAATAAAGCTTGCCTTGA | TAGTGTGTGCCCGTCTG | GAGGGATCTCTAGTTACCAGAGTCACA | GAGGGATCTCTAGTTACCAGAGTCACA |
| D-clone 1 | AAGCCTCAATAAAGCTTGCCTTGA | TAGTGTGTGCCCGTCTG | GAGGGATCTCTAGTTACCAGAGCCACA | GAGGGATCTCTAGTTACCAGAGCCACA |
| D-clone 2 | AAGCCTCAATAAAGCTTGCCTTGA | TAGTGTGTGCCCGTCTG | GAGGGATCTCTAGTTACCAGAGTCACA | GAGGGATCTCTAGTTACCAGAGTCACA |
| AE-clone 1 | AAGCCTCAATAAAGCTTGCCTTGA | TGGTGTGTGCCCGTCTG | GAGGGATCTCTAGTTACCAGAGTCCTA | GAGGGATCTCTAGTTACCAGAGTCCTA |
| AE-clone 2 | AAGCCTCAATAAAGCTTGCCTTGA | TGGTGTGTGCCCATCTG | GAGGGATCTCTAGTTACCAGAGTCCTG | GAGGGATCTCTAGTTACCAGAGTCCTG |
| F-clone | AAGCCTCAATAAAGCTTGCCTTGA | TAGTGTGTGCCCGTCTG | GAGGGATCTCTAGTTACCAGAGTCACA | GAGGGATCTCTAGTTACCAGAGTCACA |
| G-clone | AAGCCTCAATAAAGCTTGCCTTGA | TAGTGTGTGCCCGTCTG | GAGGGATCTCTAGTTACCAGAGTCACA | GAGGGATCTCTAGTTACCAGAGTCACA |
| AG-clone | AAGCCTCAATAAAGCTTGCCTTGA | TAGTGTGTGCCCGTCTG | GAGGGATCTCTAGTTACCAGAGTCACA | GAGGGATCTCTAGTTACCAGAGTCACA |

|  | JO-forward | JO-FAMZEN | JO-reverse |
| --- | --- | --- | --- |
| Primer | TACAGTGCAGGGGAAAGAATA | TTTCGGGTTTATTACAGGGACAGCAG | CTGCCCCTTCACCTTTCC |
|  |  |  |  |
| HXB2 | TACAGTGCAGGGGAAAGAATA | TTTCGGGTTTATTACAGGGACAGCAG | CTGCCCCTTCACCTTTCC |
| A-clone 1 | TACAGTGCAGGGGAAAGAATA | TTTCGGGTTTATTATAGGGACAGCAG | CTGCCCCTTCACCTTTCC |
| A-clone 2 | TACAGTGCAGGGGAAAGAATA | TTTCGGGTTTATTACAGGGACAGCAG | CTGCCCCTTCACCTTTCC |
| B-clone 1 | TACAGTGCAGGGGAAAGAATA | TTTCGGGTTTATTACAGGGACAGCAG | CTGCCCCTTCACCTTTCC |
| B-clone 2 | TACAGTGCAGGGGAAAGAATA | TTTCGGGTTTATTACAGGGACAGCAG | CTGCCCCTTCACCTTTCC |
| C-clone 1 | TACAGTGCAGGGGAAAGAATA | TTTCGGGTTTATTACAGAGACAGCAG | CTGCCCCTTCACCTTTCC |
| C-clone 2 | TACAGTGCAGGGGAAAGAATA | TTTCGGGTTTATTACAGAGACAGCAG | CTGCCCCTTCACCTTTCC |
| D-clone 1 | TACAGTGCAGGGGAAAGAATA | TTTCGGGTTTATTACAGGGACAGCAG | CTGCCCCTTCACCTTTCC |
| D-clone 2 | TACAGTGCAGGGGAAAGAATA | TTTCGGGTTTATTACAGGGACAGCAG | CTGCCCCTTCACCTTTCC |
| AE-clone 1 | TACAGTGCAGGGGAAAGAATA | TTTCGGGTTTATTACAGGGACAGCAG | CTGCCCCTTCACCTTTCC |
| AE-clone 2 | TACAGTGCAGGGGAAAGAATA | TTTCGGGTTTATTACAGGGACAGCAG | CTGCCCCTTCACCTTTCC |
| F-clone | TACAGTGCAGGGGAAAGAATA | TTCCGGGTTTATTACAGGGACAGCAG | CTGCCCCTTCACCTTTCC |
| G-clone | TACAGTGCAGGGGAAAGAATA | TTTCGGGTTTATTTCAGGGACAGCAG | CTGCCCCTTCACCTTTCC |
| AG-clone | TACAGTGCAGGGGAAAGAATA | TTTCGGGTTTATTTCAGGGACAGCAG | CTGCCCCTTCACCTTTCC |

1. **JO assay**
2. **GAG assay**

|  | GAG-forward | GAG-FAMMGB | GAG-reverse |
| --- | --- | --- | --- |
| Primer | TGGGTAAAAGTAGTAGAAGAGAAGGCTTT | TCAGCATTATCAGAAGGAG | ATGCTAAACACAGTGGGGGG |
|  |  |  |  |
| HXB2 | TGGGTAAAAGTAGTAGAAGAGAAGGCTTT | TCAGCATTATCAGAAGGAG | ATGCTAAACACAGTGGGGGG |
| A-clone 1 | TGGGTGAAAGTAATAGAAGAAAGGGGTTT | TCAGCATTATCAGAAGGAG | ATGCTGAATATAGTGGGGGG |
| A-clone 2 | TGGGTAAAGGCAATAGAAGAAAAGGCTTT | ACAGCATTATCAGAGGGAG | ATGCTAAACATAGTGGGGGG |
| B-clone 1 | TGGGTAAAAGTAGTAGAAGAAAAGGCTTT | TCAGCATTATCAGAAGGAG | ATGCTAAACACAGTGGGGGG |
| B-clone 2 | TGGGTAAAAGTAGTAGAAGAGAAGGCTTT | TCAGCATTATCAGAAGGAG | ATGCTAAACACAGTGGGGGG |
| C-clone 1 | TGGGTAAAAGTAGTAGAGGAAAAGGCTTT | ACAGCTTTATCAGAAGGAG | ATGCTAAATACAGTGGGGGA |
| C-clone 2 | TGGGTAAAAGTAATAGAGGAAAAGGCCTT | ACAGCATTATCAGAAGGAG | ATGTTAAATACAGTGGGGGG |
| D-clone 1 | TGGGTCAAAGTAATAGAAGAGAAGGCTTT | ACAGCACTATCAGAAGGAG | ATGTTAAACACAGTGGGGGG |
| D-clone 2 | TGGGTAAAAGTAATAGAGGAGAAGGCTTT | ACAGCATTATCAGAAGGAG | ATGCTAAATACAGTGGGGGG |
| AE-clone 1 | TGGGTGAAAGTAGTAGAAGAAAAGGGTTT | TCAGCATTATCAGAGGGAG | ATGCTAAATATAGTGGGGGG |
| AE-clone 2 | TGGGTGAAAGTAGTAGAAGAAAAGGGCTT | TCAGCACTATCAGAGGGAG | ATGCTAAATATAGTGGGGGG |
| F-clone | TGGGTAAAGGTGATAGAAGAGAAGGCTTT | TCAGCATTATCAGAAGGGG | ATGTTAAATACAGTGGGGGG |
| G-clone | TGGGTAAAAGTAGTAGAAGAAAAGGCCTT | TCAGCATTATCAGAAGGAG | ATGCTAAACACAGTGGGGGG |
| AG-clone | TGGGTGAAGGTAATAGAAGAGAAAGGTTT | TCAGCATTATCAGAGGGAG | ATGCTAAACATAGTGGGGGG |

**Supplementary Figure 2.** Representativeness of the subtype reference plasmids for all intra-subtype variation. Clone-specific target sequences were submitted to the LANL HIV1 Complete Nucleotide Filtered web alignment, summarized by major subtypes and CRF’s and sequence variant abundances were extracted for the subtype that each clone represents. All variants are shown as different blocks, colors indicate their genetic distance away from the (in case of two plasmids, best matching) subtype reference plasmid. Target sequences of the LTR (**A**), JO (**B**) and GAG (**C**) assays.

**A)**

**B)**

**C)**

**Supplementary Figure 3.** Primer and probe sequence complementarity of the LTR, JO and GAG assays to the LANL HIV1 Complete Nucleotide Filtered web alignment. Summarized for all sequences (**A**) and summarized for major subtypes and CRF’s (**B-D**).

**A)**

**B)**

**C)**

**D**

**Supplementary Table 8.** Quantification results of the subtype reference plasmids, numbers represent absolute copies. Coefficient of variation (CV) is shown as the percentage of standard deviation of the mean. CV’s were found to be higher in the low ranges and minute in the high ranges, and are comparable to previous reports (Zhao et al 2016, Bosman et al 2015, Carow et al 2017, Tang et al 2016, Pavsic et al 2016, Taylor et al 2015).

|  |  | **Assay** | **GAG** | | |  |  | **JO** | | |  |  | **LTR** | | |  |  |
| --- | --- | --- | --- | --- | --- | --- | --- | --- | --- | --- | --- | --- | --- | --- | --- | --- | --- |
|  |  | **Replicate** | **1** | **2** | **3** | **Mean** | **CV (%)** | **1** | **2** | **3** | **Mean** | **CV (%)** | **1** | **2** | **3** | **Mean** | **CV (%)** |
| **Dilution** | **Subtype** | **Clone** |  |  |  |  |  |  |  |  |  |  |  |  |  |  |  |
| 3125 | A | 1 | 2 | 2 | 2 | 2 | 0 | 2149 | 1988 | 2161 | 2099 | 5 | 2439 | 2422 | 2429 | 2430 | 0 |
| 3125 | A | 2 | 0 | 0 | 0 | 0 |  | 814 | 728 | 757 | 766 | 6 | 1386 | 1379 | 1344 | 1370 | 2 |
| 3125 | B | 1 | 772 | 794 | 794 | 787 | 2 | 798 | 842 | 937 | 859 | 8 | 847 | 894 | 872 | 871 | 3 |
| 3125 | B | 2 | 1089 | 890 | 1172 | 1050 | 14 | 1193 | 1250 | 1153 | 1199 | 4 | 1163 | 1220 | 1255 | 1213 | 4 |
| 3125 | C | 1 | 0 | 0 | 0 | 0 |  | 1276 | 1206 | 1171 | 1218 | 4 | 548 | 554 | 600 | 567 | 5 |
| 3125 | C | 2 | 0 | 0 | 0 | 0 |  | 548 | 540 | 594 | 561 | 5 | 850 | 826 | 807 | 828 | 3 |
| 3125 | D | 1 | 2 | 0 | 0 | 1 | 173 | 912 | 577 | 614 | 701 | 26 | 1370 | 1294 | 1330 | 1331 | 3 |
| 3125 | D | 2 | 852 | 798 | 755 | 802 | 6 | 934 | 958 | 823 | 905 | 8 | 1277 | 1259 | 1283 | 1273 | 1 |
| 3125 | AE | 1 | 0 | 0 | 0 | 0 |  | 1482 | 1412 | 1357 | 1417 | 4 | 1484 | 1583 | 1656 | 1574 | 5 |
| 3125 | AE | 2 | 0 | 0 | 0 | 0 |  | 1506 | 1511 | 1384 | 1467 | 5 | 1474 | 1447 | 1579 | 1500 | 5 |
| 3125 | F | 1 | 2 | 3 | 4 | 3 | 33 | 1638 | 1577 | 1711 | 1642 | 4 | 2225 | 2221 | 2229 | 2225 | 0 |
| 3125 | G | 1 | 1523 | 1416 | failed | 1470 | 5 | 1068 | 1020 | 1125 | 1071 | 5 | 1500 | 1577 | 1557 | 1545 | 3 |
| 3125 | AG | 1 | 0 | 0 | 2 | 1 | 173 | 751 | 754 | 745 | 750 | 1 | 2107 | 2067 | 2061 | 2078 | 1 |
|  |  | **Median** |  |  |  |  | **10** |  |  |  |  | **5** |  |  |  |  | **3** |

|  |  | **Assay** | **GAG** | | | | |  |  | **JO** | | |  |  | **LTR** | | |  |  |
| --- | --- | --- | --- | --- | --- | --- | --- | --- | --- | --- | --- | --- | --- | --- | --- | --- | --- | --- | --- |
|  |  | **Replicate** | **1** | | **2** | | **3** | **Mean** | **CV (%)** | **1** | **2** | **3** | **Mean** | **CV (%)** | **1** | **2** | **3** | **Mean** | **CV (%)** |
| **Dilution** | **Subtype** | **Clone** |  | |  | |  |  |  |  |  |  |  |  |  |  |  |  |  |
| 625 | A | 1 | 0 | | 0 | | 0 | 0 |  | 382 | 391 | 320 | 364 | 11 | 477 | 470 | 484 | 477 | 1 |
| 625 | A | 2 | 0 | | 0 | | 0 | 0 |  | 225 | 164 | 214 | 201 | 16 | 246 | 259 | 324 | 276 | 15 |
| 625 | B | 1 | 149 | | 177 | | 135 | 154 | 14 | 133 | 166 | 150 | 150 | 11 | 170 | 142 | 185 | 166 | 13 |
| 625 | B | 2 | 195 | | 228 | | 208 | 210 | 8 | 261 | 261 | 256 | 259 | 1 | 235 | 249 | 209 | 231 | 9 |
| 625 | C | 1 | 0 | | 0 | | 0 | 0 |  | 266 | 203 | 194 | 221 | 18 | 134 | 100 | 120 | 118 | 14 |
| 625 | C | 2 | 0 | | 0 | | 0 | 0 |  | 108 | 89 | 109 | 102 | 11 | 165 | 173 | 151 | 163 | 7 |
| 625 | D | 1 | 0 | | 0 | | 0 | 0 |  | 177 | 104 | 125 | 135 | 28 | 311 | 251 | 281 | 281 | 11 |
| 625 | D | 2 | 167 | | 169 | | 182 | 173 | 5 | 174 | 164 | 194 | 177 | 9 | 311 | 257 | 306 | 291 | 10 |
| 625 | AE | 1 | 2 | | 0 | | 0 | 1 | 173 | 253 | 253 | 293 | 266 | 9 | 275 | 317 | 271 | 288 | 9 |
| 625 | AE | 2 | 0 | | 0 | | 0 | 0 |  | 327 | 245 | 336 | 303 | 17 | 267 | 319 | 269 | 285 | 10 |
| 625 | F | 1 | 0 | | 0 | | 0 | 0 |  | 341 | 368 | 357 | 355 | 4 | 403 | 434 | 471 | 436 | 8 |
| 625 | G | 1 | 316 | | 292 | | 327 | 312 | 6 | 219 | 263 | 241 | 241 | 9 | 296 | 304 | 345 | 315 | 8 |
| 625 | AG | 1 | 0 | | 0 | | 0 | 0 |  | 113 | 90 | 96 | 100 | 12 | 402 | 451 | 395 | 416 | 7 |
|  |  | **Median** |  | |  | |  |  | **8** |  |  |  |  | **11** |  |  |  |  | **9** |
|  |  |  |  | |  | |  |  |  |  |  |  |  |  |  |  |  |  |  |
| 125 | A | 1 | 0 | | 0 | | 0 | 0 |  | 59 | 93 | 75 | 76 | 22 | 70 | 92 | 94 | 85 | 16 |
| 125 | A | 2 | 0 | | 0 | | 0 | 0 |  | 32 | 23 | 42 | 32 | 29 | 53 | 61 | 68 | 61 | 12 |
| 125 | B | 1 | 23 | | 32 | | 34 | 30 | 20 | 31 | 25 | 30 | 29 | 11 | 30 | 33 | 23 | 29 | 18 |
| 125 | B | 2 | 44 | | 62 | | 50 | 52 | 18 | 29 | 38 | 50 | 39 | 27 | 47 | 45 | 50 | 47 | 5 |
| 125 | C | 1 | 2 | | 0 | | 0 | 1 | 173 | 32 | 32 | 46 | 37 | 22 | 12 | 5 | 20 | 12 | 61 |
| 125 | C | 2 | 0 | | 0 | | 0 | 0 |  | 23 | 17 | 21 | 20 | 15 | 25 | 26 | 30 | 27 | 10 |
| 125 | D | 1 | 0 | | 0 | | 0 | 0 |  | 30 | 35 | 23 | 29 | 21 | 48 | 42 | 67 | 52 | 25 |
| 125 | D | 2 | 32 | | 29 | | 23 | 28 | 16 | 48 | 41 | 33 | 41 | 18 | 58 | 43 | 51 | 51 | 15 |
| 125 | AE | 1 | 0 | | 0 | | 0 | 0 |  | 61 | 42 | 76 | 60 | 29 | 65 | 62 | 67 | 65 | 4 |
| 125 | AE | 2 | 0 | | 0 | | 0 | 0 |  | 74 | 64 | 60 | 66 | 11 | 41 | 74 | 62 | 59 | 28 |
| 125 | F | 1 | 0 | | 0 | | 0 | 0 |  | 65 | 83 | 58 | 69 | 19 | 90 | 77 | 87 | 85 | 8 |
| 125 | G | 1 | 76 | | 53 | | 63 | 64 | 18 | 37 | 51 | 33 | 40 | 23 | 39 | 55 | 70 | 55 | 28 |
| 125 | AG | 1 | 0 | | 2 | | 0 | 1 | 173 | 17 | 18 | 15 | 17 | 9 | 72 | 113 | 108 | 98 | 23 |
|  |  | **Median** |  | |  | |  |  | **19** |  |  |  |  | **21** |  |  |  |  | **16** |
|  |  | **Assay** | **GAG** | | | | |  |  | **JO** | | |  |  | **LTR** | | |  |  |
|  |  | **Replicate** | **1** | **2** | | **3** | | **Mean** | **CV (%)** | **1** | **2** | **3** | **Mean** | **CV (%)** | **1** | **2** | **3** | **Mean** | **CV (%)** |
| **Dilution** | **Subtype** | **Clone** |  |  | |  | |  |  |  |  |  |  |  |  |  |  |  |  |
| 25 | A | 1 | 0 | 0 | | 0 | | 0 |  | 9 | 18 | 15 | 14 | 33 | 14 | 16 | 9 | 13 | 28 |
| 25 | A | 2 | 0 | 0 | | 0 | | 0 |  | 12 | 8 | 5 | 8 | 42 | 8 | 15 | 13 | 12 | 30 |
| 25 | B | 1 | 2 | 3 | | 6 | | 4 | 57 | 8 | 2 | 2 | 4 | 87 | 4 | 2 | 3 | 3 | 33 |
| 25 | B | 2 | 7 | 8 | | 10 | | 8 | 18 | 13 | 13 | 7 | 11 | 31 | 10 | 3 | 12 | 8 | 57 |
| 25 | C | 1 | 0 | 0 | | 0 | | 0 |  | 2 | 0 | 6 | 3 | 115 | 2 | 0 | 4 | 2 | 100 |
| 25 | C | 2 | 0 | 0 | | 0 | | 0 |  | 2 | 8 | 8 | 6 | 58 | 8 | 7 | 3 | 6 | 44 |
| 25 | D | 1 | 0 | 0 | | 0 | | 0 |  | 6 | 8 | 8 | 7 | 16 | 11 | 9 | 12 | 11 | 14 |
| 25 | D | 2 | 0 | 6 | | 4 | | 3 | 92 | 5 | 8 | 4 | 6 | 37 | 6 | 14 | 11 | 10 | 39 |
| 25 | AE | 1 | 0 | 0 | | 0 | | 0 |  | 16 | 8 | 10 | 11 | 37 | 6 | 11 | 18 | 12 | 52 |
| 25 | AE | 2 | 0 | 3 | | 3 | | 2 | 87 | 10 | 14 | 6 | 10 | 40 | 14 | 19 | 13 | 15 | 21 |
| 25 | F | 1 | 0 | 0 | | 0 | | 0 |  | 11 | 10 | 9 | 10 | 10 | 12 | 17 | 20 | 16 | 25 |
| 25 | G | 1 | 7 | 11 | | 20 | | 13 | 53 | 14 | 5 | 8 | 9 | 51 | 9 | 7 | 21 | 12 | 61 |
| 25 | AG | 1 | 0 | 0 | | 0 | | 0 |  | 4 | 2 | 2 | 3 | 43 | 15 | 16 | 17 | 16 | 6 |
|  |  | **Median** |  |  | |  | |  | **57** |  |  |  |  | **40** |  |  |  |  | **33** |
|  |  |  |  |  | |  | |  |  |  |  |  |  |  |  |  |  |  |  |
| 5 | A | 1 | 0 | 0 | | 0 | | 0 |  | 0 | 2 | 3 | 2 | 92 | 2 | 2 | 3 | 2 | 25 |
| 5 | A | 2 | 0 | 0 | | 0 | | 0 |  | 4 | 4 | 3 | 4 | 16 | 2 | 4 | 0 | 2 | 100 |
| 5 | B | 1 | 3 | 0 | | 0 | | 1 | 173 | 0 | 1 | 0 | 0 | 173 | 2 | 0 | 5 | 2 | 108 |
| 5 | B | 2 | 4 | 0 | | 4 | | 3 | 87 | 5 | 5 | 2 | 4 | 43 | 3 | 0 | 0 | 1 | 173 |
| 5 | C | 1 | 0 | 0 | | 0 | | 0 |  | 9 | 7 | 4 | 7 | 38 | 0 | 2 | 0 | 1 | 173 |
| 5 | C | 2 | 0 | 0 | | 0 | | 0 |  | 9 | 0 | 6 | 5 | 92 | 0 | 0 | 0 | 0 |  |
| 5 | D | 1 | 0 | 0 | | 0 | | 0 |  | 2 | 2 | 2 | 2 | 0 | 2 | 2 | 0 | 1 | 87 |
| 5 | D | 2 | 2 | 0 | | 0 | | 1 | 173 | 2 | 2 | 2 | 2 | 0 | 4 | 0 | 0 | 1 | 173 |
| 5 | AE | 1 | 0 | 0 | | 0 | | 0 |  | 3 | 5 | 3 | 4 | 31 | 0 | 2 | 5 | 2 | 108 |
| 5 | AE | 2 | 0 | 0 | | 0 | | 0 |  | 2 | 2 | 2 | 2 | 0 | 2 | 4 | 2 | 3 | 43 |
| 5 | F | 1 | 0 | 0 | | 0 | | 0 |  | 2 | 3 | 5 | 3 | 46 | 8 | 6 | 2 | 5 | 57 |
| 5 | G | 1 | 4 | 0 | | 0 | | 1 | 173 | 2 | 4 | 0 | 2 |  | 2 | 0 | 8 | 3 | 125 |
| 5 | AG | 1 | 0 | 0 | | 0 | | 0 |  | 0 | 0 | 0 | 0 |  | 3 | 3 | 2 | 3 | 22 |
|  |  | **Median** |  |  | |  | |  | **173** |  |  |  |  | **38** |  |  |  |  | **104** |
|  |  | **Assay** | **GAG** | | | | |  |  | **JO** | | |  |  | **LTR** | | |  |  |
|  |  | **Replicate** | **1** | | **2** | | **3** | **Mean** | **CV (%)** | **1** | **2** | **3** | **Mean** | **CV (%)** | **1** | **2** | **3** | **Mean** | **CV (%)** |
| **Dilution** | **Subtype** | **Clone** |  | |  | |  |  |  |  |  |  |  |  |  |  |  |  |  |
| 1 | A | 1 | 0 | | 0 | | 0 | 0 |  | 0 | 2 | 0 | 1 | 173 | 0 | 0 | 0 | 0 |  |
| 1 | A | 2 | 0 | | 0 | | 0 | 0 |  | 0 | 0 | 0 | 0 |  | 2 | 3 | 0 | 2 | 92 |
| 1 | B | 1 | 0 | | 0 | | 0 | 0 |  | 0 | 0 | 0 | 0 |  | 0 | 0 | 0 | 0 |  |
| 1 | B | 2 | 0 | | 0 | | 0 | 0 |  | 0 | 0 | 0 | 0 |  | 2 | 0 | 0 | 1 | 173 |
| 1 | C | 1 | 0 | | 0 | | 0 | 0 |  | 0 | 0 | 0 | 0 |  | 0 | 0 | 0 | 0 |  |
| 1 | C | 2 | 0 | | 0 | | 0 | 0 |  | 2 | 0 | 0 | 1 | 173 | 0 | 0 | 2 | 1 | 173 |
| 1 | D | 1 | 0 | | 0 | | 0 | 0 |  | 0 | 0 | 2 | 1 | 173 | 0 | 0 | 0 | 0 |  |
| 1 | D | 2 | 0 | | 0 | | 0 | 0 |  | 0 | 0 | 0 | 0 |  | 0 | 0 | 3 | 1 | 173 |
| 1 | AE | 1 | 0 | | 0 | | 0 | 0 |  | 0 | 0 | 2 | 1 | 173 | 0 | 3 | 0 | 1 | 173 |
| 1 | AE | 2 | 0 | | 0 | | 0 | 0 |  | 0 | 3 | 0 | 1 | 173 | 0 | 0 | 0 | 0 |  |
| 1 | F | 1 | 0 | | 0 | | 0 | 0 |  | 0 | 0 | 2 | 1 | 173 | 0 | 0 | 4 | 1 | 173 |
| 1 | G | 1 | 2 | | 0 | | 0 | 1 | 173 | 0 | 0 | 0 | 0 |  | 0 | 0 | 0 | 0 |  |
| 1 | AG | 1 | 0 | | 2 | | 0 | 1 | 173 | 0 | 0 | 0 | 0 |  | 5 | 0 | 2 | 2 | 108 |
|  |  | **Median** |  | |  | |  |  | **173** |  |  |  |  | **173** |  |  |  |  | **173** |
|  |  |  |  | |  | |  |  |  |  |  |  |  |  |  |  |  |  |  |
| 0.2 | A | 1 | 0 | | 0 | | 0 | 0 |  | 0 | 0 | 0 | 0 |  | 0 | 0 | 0 | 0 |  |
| 0.2 | A | 2 | 0 | | 0 | | 0 | 0 |  | 0 | 0 | 0 | 0 |  | 0 | 0 | 2 | 1 | 173 |
| 0.2 | B | 1 | 0 | | 2 | | 0 | 1 | 173 | 0 | 2 | 0 | 1 | 173 | 0 | 0 | 0 | 0 |  |
| 0.2 | B | 2 | 0 | | 0 | | 0 | 0 |  | 0 | 0 | 0 | 0 |  | 0 | 0 | 0 | 0 |  |
| 0.2 | C | 1 | 0 | | 2 | | 0 | 1 | 173 | 0 | 0 | 0 | 0 |  | 0 | 0 | 0 | 0 |  |
| 0.2 | C | 2 | 0 | | 0 | | 0 | 0 |  | 4 | 4 | 4 | 4 | 0 | 0 | 2 | 0 | 1 | 173 |
| 0.2 | D | 1 | 0 | | 0 | | 0 | 0 |  | 0 | 0 | 0 | 0 |  | 0 | 0 | 0 | 0 |  |
| 0.2 | D | 2 | 0 | | 0 | | 0 | 0 |  | 0 | 0 | 0 | 0 |  | 2 | 0 | 0 | 1 | 173 |
| 0.2 | AE | 1 | 0 | | 0 | | 0 | 0 |  | 0 | 0 | 0 | 0 |  | 0 | 0 | 2 | 1 | 173 |
| 0.2 | AE | 2 | 0 | | 5 | | 0 | 2 | 173 | 0 | 0 | 0 | 0 |  | 0 | 0 | 0 | 0 |  |
| 0.2 | F | 1 | 0 | | 0 | | 0 | 0 |  | 0 | 2 | 0 | 1 | 173 | 0 | 0 | 0 | 0 |  |
| 0.2 | G | 1 | 0 | | 0 | | 0 | 0 |  | 0 | 0 | 0 | 0 |  | 0 | 0 | 0 | 0 |  |
| 0.2 | AG | 1 | 0 | | 0 | | 0 | 0 |  | 0 | 0 | 0 | 0 |  | 0 | 0 | 0 | 0 |  |
|  |  | **Median** |  | |  | |  |  | **173** |  |  |  |  | **173** |  |  |  |  | **173** |

**Supplementary Table 9.** Quantification results of the patient isolates, numbers represent absolute copies.

|  | **Assay** | **GAG** | | |  |  | **JO** | | |  |  | **LTR** | | |  |  |
| --- | --- | --- | --- | --- | --- | --- | --- | --- | --- | --- | --- | --- | --- | --- | --- | --- |
|  | **Replicate** | **1** | **2** | **3** | **Mean** | **CV (%)** | **1** | **2** | **3** | **Mean** | **CV (%)** | **1** | **2** | **3** | **Mean** | **CV (%)** |
| **Patient ID** | **Subtype** |  |  |  |  |  |  |  |  |  |  |  |  |  |  |  |
| A-patient 1 | A | 1 | 0 | 0 | 0 | 173 | 21 | 14 | 26 | 20 | 30 | 51 | 38 | 47 | 45 | 15 |
| A-patient 2 | A | 0 | 0 | 0 | 0 |  | 732 | 770 | 742 | 748 | 3 | 1500 | 1430 | 1448 | 1459 | 2 |
| A-patient 3 | A | 0 | 2 | 0 | 1 | 173 | 213 | 221 | 163 | 199 | 16 | 361 | 302 | 306 | 323 | 10 |
| A-patient 4 | A | 0 | 0 | 2 | 1 | 173 | 2 | 3 | 5 | 3 | 46 | 9 | 5 | 11 | 8 | 37 |
| B-patient 1 | B | 2 | 0 | 0 | 1 | 173 | 1006 | 1085 | 911 | 1001 | 9 | 2514 | 2489 | 2638 | 2547 | 3 |
| B-patient 2 | B | 62 | 56 | 71 | 63 | 12 | 67 | 51 | 68 | 62 | 15 | 167 | 136 | 162 | 155 | 11 |
| B-patient 3 | B | 966 | 978 | 996 | 980 | 2 | 1166 | 1084 | 1306 | 1185 | 9 | 2252 | 1836 | failed | 2044 | 14 |
| B-patient 4 | B | 80 | 44 | 44 | 56 | 37 | 77 | 60 | 78 | 72 | 14 | 114 | 134 | 123 | 124 | 8 |
| B-patient 5 | B | 37 | 32 | 41 | 37 | 12 | 34 | 29 | 23 | 29 | 19 | 52 | 73 | 69 | 65 | 17 |
| C-patient 1 | C | 8 | 7 | 0 | 5 | 87 | 46 | 41 | 51 | 46 | 11 | 126 | 129 | 131 | 129 | 2 |
| C-patient 2 | C | 3 | 0 | 0 | 1 | 173 | 203 | 190 | 232 | 208 | 10 | 556 | 505 | 535 | 532 | 5 |
| C-patient 3 | C | 0 | 0 | 0 | 0 |  | 122 | 103 | 113 | 113 | 8 | 225 | 231 | 219 | 225 | 3 |
| D-patient 1 | D | 4 | 2 | 0 | 2 | 100 | 9 | 9 | 11 | 10 | 12 | 28 | 40 | 34 | 34 | 18 |
| D-patient 2 | D | 29 | 21 | 26 | 25 | 16 | 76 | 45 | 64 | 62 | 25 | 174 | 191 | 179 | 181 | 5 |
| AE-patient 1 | AE | 0 | 0 | 0 | 0 |  | 45 | 43 | 57 | 48 | 16 | 89 | 67 | 100 | 85 | 20 |
| AE-patient 2 | AE | 0 | 0 | 0 | 0 |  | 57 | 66 | 56 | 60 | 9 | 169 | 172 | 141 | 161 | 11 |
| AE-patient 3 | AE | 0 | 0 | 0 | 0 |  | 5 | 8 | 8 | 7 | 25 | 17 | 26 | 13 | 19 | 36 |
| AE-patient 4 | AE | 0 | 0 | 0 | 0 |  | 95 | 83 | 95 | 91 | 8 | 227 | 221 | 244 | 231 | 5 |
| F-patient 1 | F | 0 | 0 | 0 | 0 |  | 143 | 144 | 121 | 136 | 10 | 207 | 257 | 217 | 227 | 12 |
| F-patient 2 | F | 2 | 0 | 0 | 1 | 173 | 7 | 5 | 11 | 8 | 40 | 8 | 5 | 6 | 6 | 24 |
| F-patient 3 | F | 0 | 0 | 0 | 0 |  | 59 | 46 | 57 | 54 | 13 | 84 | 114 | 95 | 98 | 16 |
| F-patient 4 | F | 0 | 0 | 0 | 0 |  | 16 | 10 | 16 | 14 | 25 | 26 | 13 | 26 | 22 | 35 |
| G-patient 1 | G | 0 | 0 | 0 | 0 |  | 16 | 19 | 17 | 17 | 9 | 53 | 55 | 40 | 49 | 17 |
| G-patient 2 | G | 0 | 0 | 0 | 0 |  | 31 | 38 | 37 | 35 | 11 | 76 | 65 | 89 | 77 | 16 |
| G-patient 3 | G | 5 | 0 | 10 | 5 | 100 | 11 | 7 | 8 | 9 | 24 | 10 | 19 | 24 | 18 | 40 |
| AG-patient 1 | AG | 2 | 0 | 0 | 1 | 173 | 77 | 55 | 71 | 68 | 17 | 94 | 139 | 100 | 111 | 22 |
| AG-patient 2 | AG | 0 | 0 | 0 | 0 |  | 95 | 64 | 60 | 73 | 26 | 296 | 260 | 294 | 283 | 7 |
| AG-patient 3 | AG | 0 | 0 | 0 | 0 |  | 0 | 3 | 5 | 3 | 94 | 9 | 19 | 17 | 15 | 35 |
| AG-patient 4 | AG | 0 | 0 | 0 | 0 |  | 6 | 4 | 2 | 4 | 50 | 145 | 155 | 178 | 159 | 11 |
|  | **Median** |  |  |  |  | 100 |  |  |  |  | 14 |  |  |  |  | 15 |

Supplementary Figure 4. Droplet plots of PBMC DNA and water NTCs. Negative droplets in black, ddpcRquant threshold in blue and positive droplets in red. False-positive rates are comparable to other reports showing that digital PCR suffers from stochastic false-positives (Henrich et al. 2012, Strain et al. 2013, Bosman et al. 2015, Kiselinova et al. 2014, Trypsteen et al. 2015, Vynkc et al. 2016, Rutsaert et al. 2018).


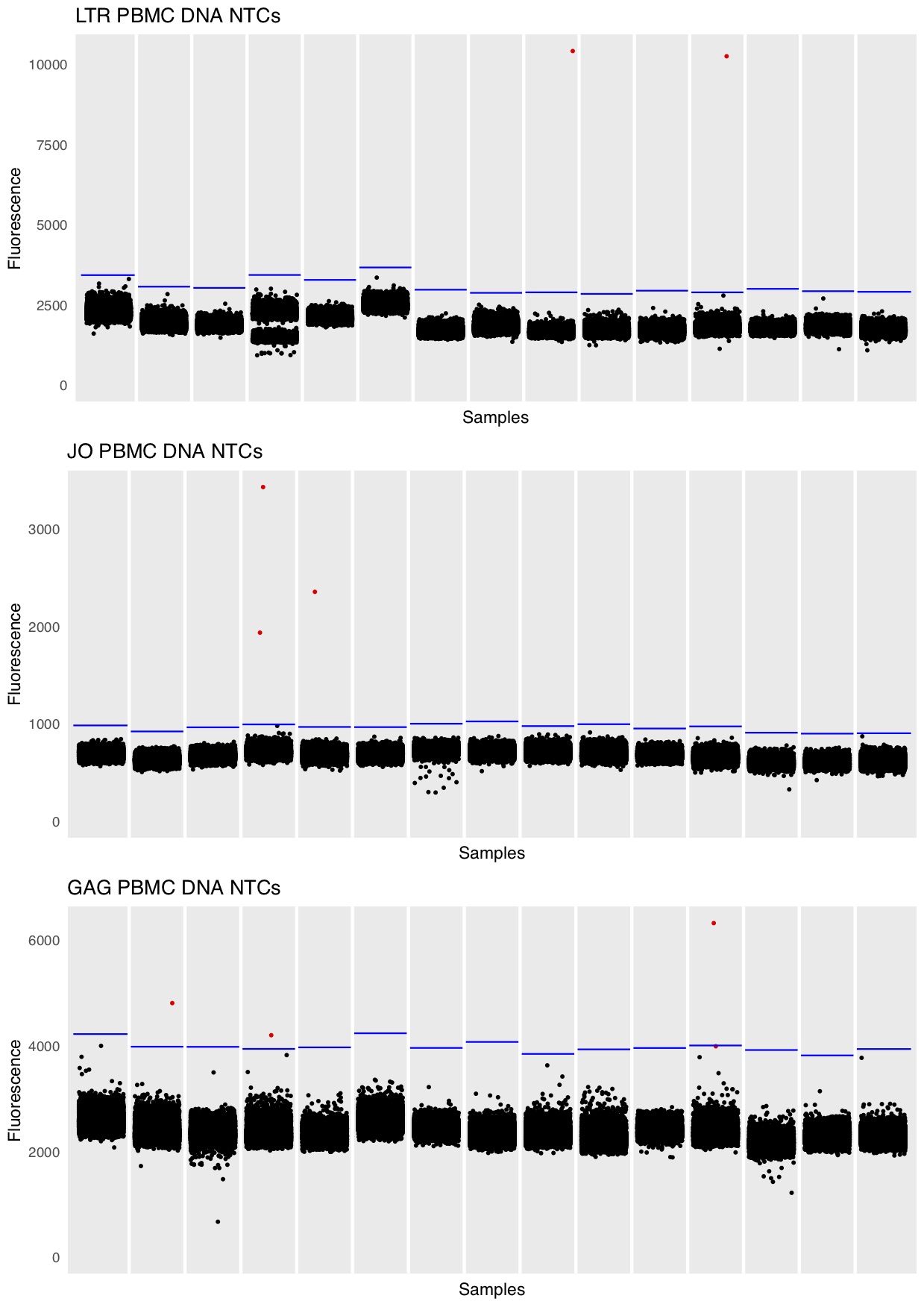


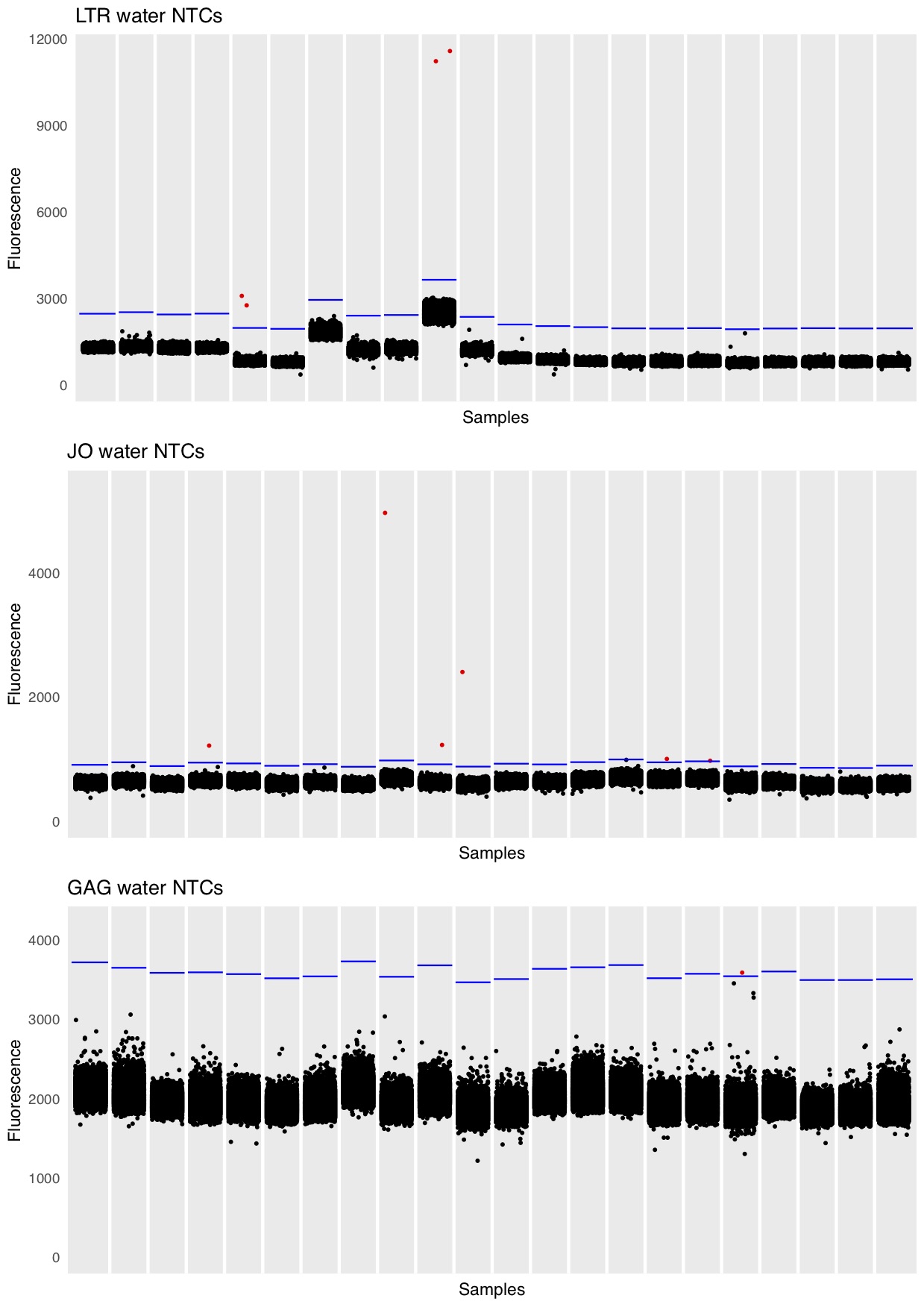


**Supplementary Figure 5.** Droplet distributions of detectable LTR, JO and GAG results in the 3125 concentration subtype reference plasmid experiments. Droplets are red or black to indicate positive or negative fraction, after application of ddpcRquant thresholds. Blue line depicts median of positive fraction.


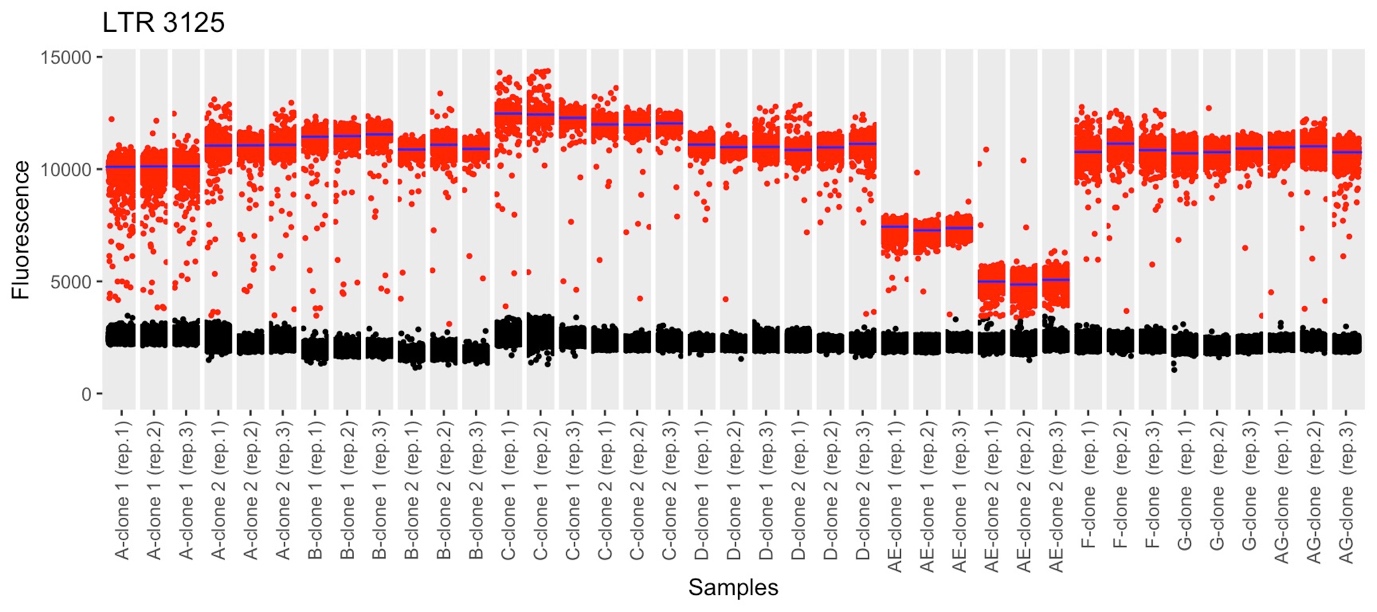


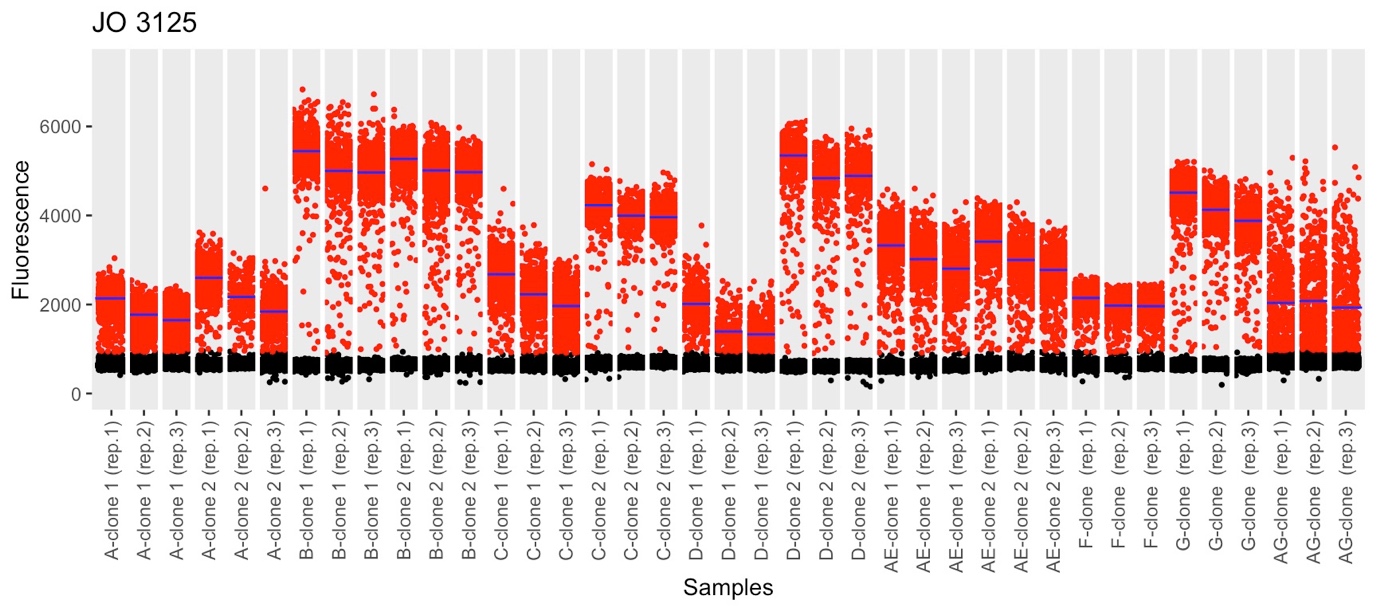


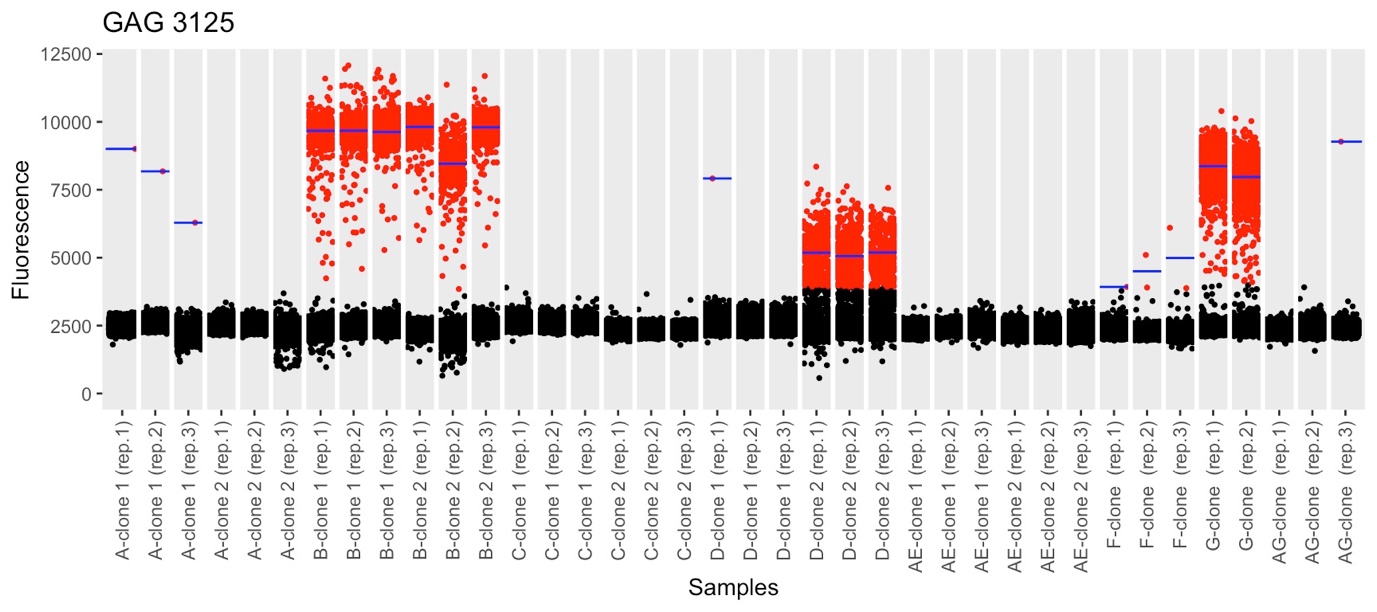


**Supplementary Figure 6.** Correlation between LTR and JO positive cloud difference (X-axis) and LTR and JO quantification results difference (Y-axis). Red line shows robust regression (p = 0.011).


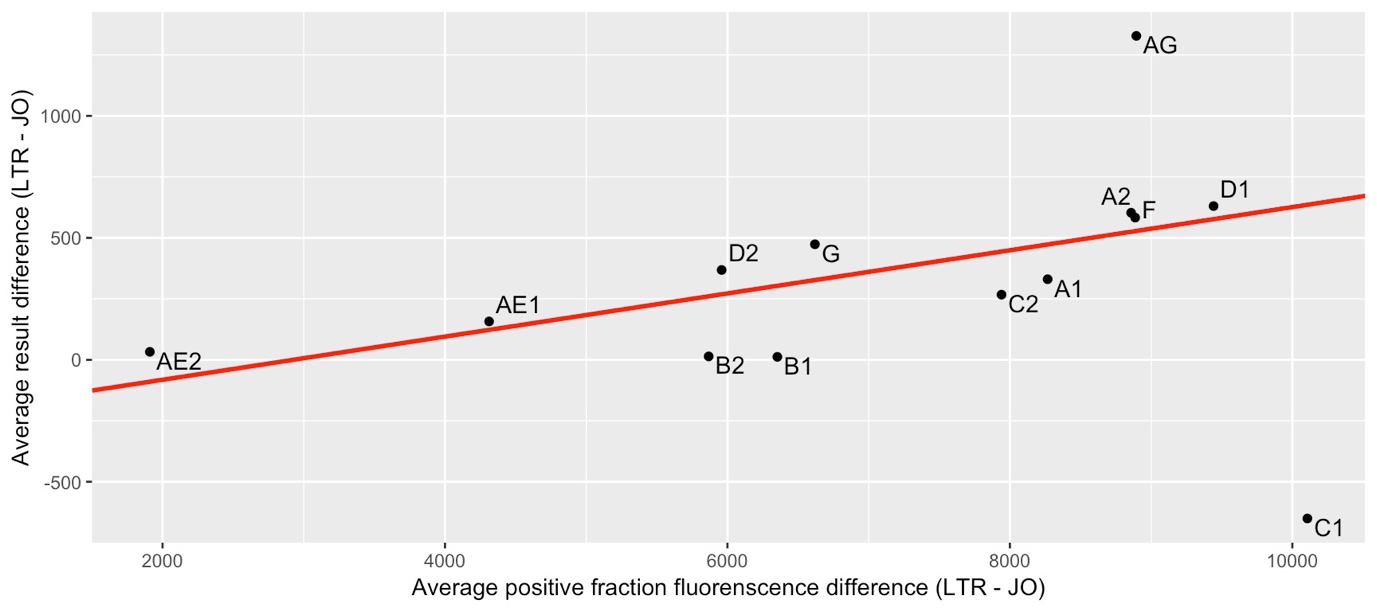


**Supplementary Text 2.** On the causes of cloud separation distance.

Interestingly, superior quantification outcome of the LTR assay cannot be attributed to superior complementarity to the subtype panel as the JO assay was found to bear fewer mismatches than the LTR assay. This leads to suspect that contrary to detectability, cloud separation distance and quantification outcome may be affected by factors other than target sequence complementarity. Amplicon length has previously been reported to inversely correlate with PCR efficiency and in our study inversely correlates with median intensity of the positive fraction of plasmid droplet distributions (amplicon lengths of 80, 115 and 165 and median fluorescence of 10967, 8179 and 2806 for LTR, GAG and JO, respectively) [1–3]. In addition, the choice of quencher was found to affect maximum fluorescence levels and cloud separation distances of LTR and JO assays when tested against U1 positive control DNA (Supplementary Figure 7). Although both quenchers produce comparable results in both assays, ZEN-quenched probes display a lower fluorescence intensity of the positive fraction as well as the negative fraction in both assays. The effect on the positive fractions of both assays is comparable and suggests that the more powerful ZEN-quencher exhibits residual quenching even after the physical link is broken. The effect on the negative fraction is especially notable for the JO assay, suggesting that boosted quenching of the internal ZEN-quencher may be especially beneficial for the 26-nucleotide JO-probe where the 5’ reporter is distant from the 3’ quencher, whereas this type of quencher is not required for the 17-nucleotide LTR probe. Because cloud separation distance was found to be greatest in case of an MGB-quencher for LTR and in case of a ZEN-quencher for JO, configurations were chosen accordingly in order to provide the assays with the largest possible buffer for mismatch-induced loss of PCR efficiency. From our experimental setup with only limited probe-quencher combinations it however remains elusive under which conditions and to what extent cloud separation distance and subsequent quantification outcome is affected by complementarity, amplicon length, probe length and quencher and additional factors that have previously been reported to affect PCR efficiency such as melting temperature, primer length, primer GC content, amplicon GC content, inhibitors and secondary structures [3–8].

1. Debode F, Marien A, Janssen E, Bragard C, Berben G. Influence of the amplicon length on real-time PCR results. Biotechnol Agron Société Environ. 2017;21(1):3–11.

2. Kong H, Zhu M, Cui F, Wang S, Gao X, Lu S, et al. Quantitative assessment of short amplicons in FFPE-derived long-chain RNA. Sci Rep. 2014;4(1):7246.

3. Mallona I, Weiss J, Egea-Cortines M. pcrEfficiency: a Web tool for PCR amplification efficiency prediction. BMC Bioinformatics. 2011;12(1):404.

4. Sedlak RH, Kuypers J, Jerome KR. A multiplexed droplet digital PCR assay performs better than qPCR on inhibition prone samples. Diagn Microbiol Infect Dis. 2014;80(4):285–6.

5. Dingle TC, Sedlak RH, Cook L, Jerome KR. Tolerance of droplet-digital PCR vs real-time quantitative PCR to inhibitory substances. Clin Chem. 2013;59(11):1670–2.

6. Hall Sedlak R, Jerome KR. The potential advantages of digital PCR for clinical virology diagnostics. Expert Rev Mol Diagn. 2014;14(4):501–7.

7. Bustin S, Huggett J. qPCR primer design revisited. Biomol Detect Quantif. 2017;14(March):19–28.

8. Stevenson J, Hymas W, Hillyard D. Effect of sequence polymorphisms on performance of two real-time PCR assays for detection of herpes simplex virus. J Clin Microbiol. 2005;43(5):2391–8.

**Supplementary Figure 7.** Effect of different quenchers on droplet distributions. Depicted are two U1 DNA samples of the LTR and JO assays with FAM-labelled probes with different quenchers: LTR with an MGB-quencher, LTR with a ZEN-quencher, JO with an MGB-quencher and JO with a ZEN-quencher.


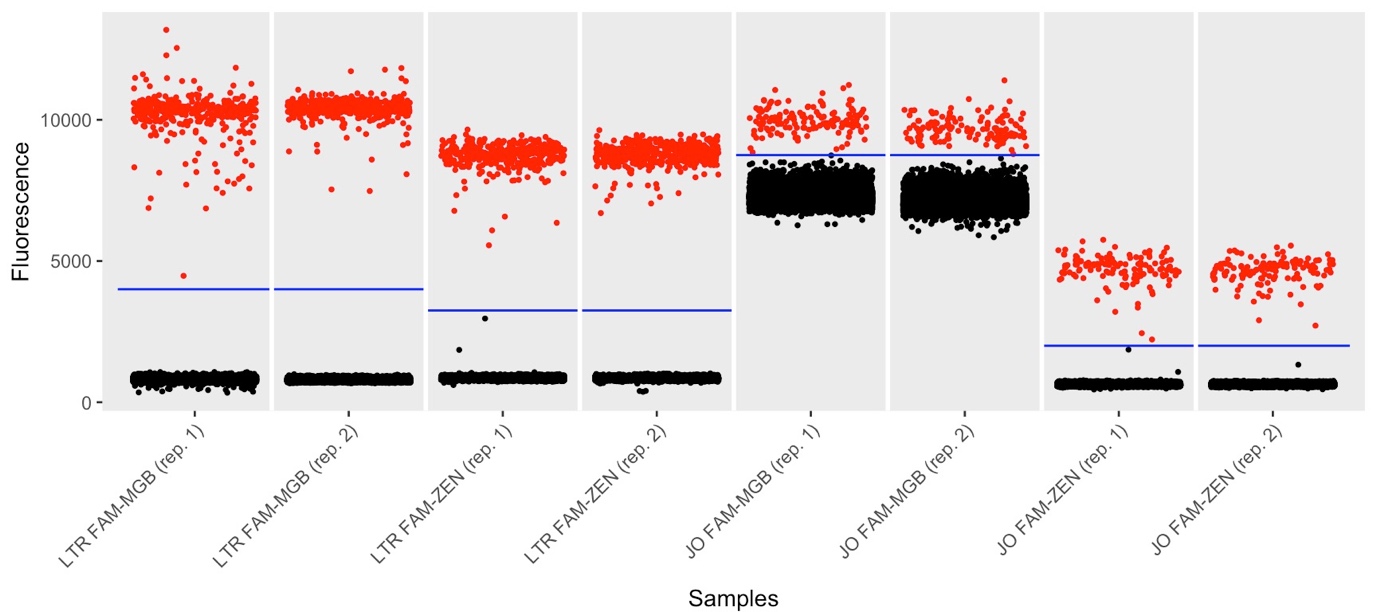

Supplement: Supplementary file 1 — Table S1. Virus isolates obtained from BBI Biotech Research Laboratories Inc. used for cloning the subtype reference plasmids. Table S2. Primers used for cloning the subtype reference plasmids. Table S3. RT‐PCR cycling parameters used for subtype reference plasmid cloning. Table S4. Nested PCR cycling parameters used for subtype reference plasmid cloning. Data S1. Threshold‐setting considerations. Table S5. Numbers of false‐positive water NTCs depending on threshold algorithm out of 22 water NTCs tested per assay. Table S6. Numbers of positive PBMC DNA NTCs depending on threshold algorithm out of 15 PBMC DNA NTCs tested per assay. Figure S1. Primer and probe sequence complementarity of our assays and other assays found in the literature to LANL HIV1 Complete Nucleotide Filtered web alignment Table S7. Complementarity of the LTR (A), JO (B) and GAG (C) primers and probes to their binding sites in the subtype reference plasmids Figure S2. Representativeness of the subtype reference plasmids for all intra‐subtype variation Figure S3. Primer and probe sequence complementarity of the LTR, JO and GAG assays to the LANL HIV1 Complete Nucleotide Filtered web alignment Table S8. Quantification results of the subtype reference plasmids; numbers represent absolute copies Table S9. Quantification results of the patient isolates; numbers represent absolute copies Figure S4. Droplet plots of PBMC DNA and water NTCs Figure S5. Droplet distributions of detectable LTR, JO and GAG results in the 3125 concentration subtype reference plasmid experiments Figure S6. Correlation between LTR‐ and JO‐positive cloud difference (X‐axis) and LTR and JO quantification results difference (Y‐axis) Data S2. On the causes of cloud separation distance. Figure S7. Effect of different quenchers on droplet distributions. [file JIA2-21-e25185-s001.docx]
